# *Ex vivo* modelling of cardiac injury identifies ferroptosis-related pathways as a potential therapeutic avenue for translational medicine

**DOI:** 10.1016/j.yjmcc.2024.09.012

**Published:** 2024-09-26

**Authors:** Naisam Abbas, Marco Bentele, Florian J.G. Waleczek, Maximilian Fuchs, Annette Just, Angelika Pfanne, Andreas Pich, Sophie Linke, Susanne Neumüller, Angelika Stucki-Koch, Maria Jordan, Filippo Perbellini, Christopher Werlein, Wilhelm Korte, Fabio Ius, Arjang Ruhparwar, Natalie Weber, Jan Fiedler, Thomas Thum

**Affiliations:** a Institute of Molecular and Translational Therapeutic Strategies (IMTTS), Hannover Medical School, Hannover, Germany; b Fraunhofer Institute of Toxicology and Experimental Medicine (ITEM), Hannover, Germany; c Fraunhofer Cluster of Excellence Immune-Mediated Diseases (CIMD), Hannover, Germany; d Institute of Toxicology and Core Unit Proteomics, Hannover Medical School, Hannover, Germany; e Institute of Pathology, Hannover Medical School, Hannover, Germany; f Department of Cardiothoracic, Transplantation and Vascular Surgery, Hannover Medical School, Hannover, Germany; g Center for Translational Regenerative Medicine, Hannover Medical School, Hannover, Germany

**Keywords:** Cardiac injury, Living myocardial slice, Ferrostatin, Cardiac fibroblast, Metallothionein

## Abstract

**Background:**

Heart failure (HF) is a burgeoning health problem worldwide. Often arising as a result of cardiac injury, HF has become a major cause of mortality with limited availability of effective treatments. Ferroptotic pathways, triggering an iron-dependent form of cell death, are known to be potential key players in heart disease. This form of cell death does not exhibit typical characteristics of programmed cell death, and is mediated by impaired iron metabolism and lipid peroxidation signalling.

**Objectives:**

The aim of this study is to establish an *ex-vivo* model of myocardial injury in living myocardial slices (LMS) and to identify novel underlying mechanisms and potential therapeutic druggable target(s).

**Methods and results:**

In this study, we employed LMS as an *ex vivo* model of cardiac injury to investigate underlying mechanisms and potential therapeutic targets. Cryoinjury was induced in adult rat LMS, resulting in 30 % tissue damage. Cryoinjured LMS demonstrated impaired contractile function, cardiomyocyte hypertrophy, inflammation, and cardiac fibrosis, closely resembling *in vivo* cardiac injury characteristics. Proteomic analysis revealed an enrichment of factors associated with ferroptosis in the injured LMS, suggesting a potential causative role. To test this hypothesis, we pharmacologically inhibited ferroptotic pathways using ferrostatin (Fer-1) in the cryoinjured rat LMS, resulting in attenuation of structural changes and repression of pro-fibrotic processes. Furthermore, LMS generated from failing human hearts were used as a model of chronic heart failure. In this model, Fer-1 treatment was observed to reduce the expression of ferroptotic genes, enhances contractile function and improves tissue viability. Blocking ferroptosis-associated pathways in human cardiac fibroblasts (HCFs) resulted in a downregulation of fibroblast activation genes, a decrease in fibroblast migration capacity, and a reduction in reactive oxygen species production. RNA sequencing analysis of Fer-1-treated human LMS implicated metallothioneins as a potential underlying mechanism for the inhibition of these pathways. This effect is possibly mediated through the replenishment of glutathione reserves.

**Conclusions:**

Our findings highlight the potential of targeting ferroptosis-related pathways and metallothioneins as a promising strategy for the treatment of heart disease.

## 1 Introduction

Cardiovascular diseases (CVD) pose a substantial global health challenge, representing a leading cause of morbidity and mortality worldwide [1]. Cardiac injury significantly contributes to the development of certain cardiovascular diseases that can have long-term consequences, including the progression to heart failure. Myocardial injury can occur as a result of various pathophysiological processes, which can be categorized into two main groups: ischemic and non-ischemic causes [2]. Following cardiac injury, non-contractile myocardium develops, triggering pathological compensatory mechanisms in the remaining viable tissue. The maladaptive response to cardiac injury results in gradual remodelling of the heart, which often contributes to the onset of heart failure. Current treatments for heart failure aim to minimize the adverse remodelling processes and improve long-term outcomes. Nevertheless, the urgency to develop innovative therapies is evident, prompting ongoing research to explore new avenues in order to meet this demand [3].

Of particular interest in the respect of cardiac remodelling are hypertrophy and fibrosis, which are tightly interwoven and mutually trigger each other [4]. Cardiac hypertrophy is a classic adaptive response to overloads induced by cardiomyocyte (CM) loss during injury and disease. The increasing muscle mass assumes a key role in the compensation for hemodynamic stress. This increase in mass is due to the hypertrophy of existing myocytes rather than hyperplasia, due to CMs' limited capacity for replication after birth [5]. In the context of cardiac remodelling, cardiac fibrosis plays an essential role and is characterized by fibroblast accumulation and increased deposition of extracellular matrix (ECM) [6]. In disease conditions, cardiac fibroblasts become activated and differentiate to myofibroblasts, which are characterized by expressing high levels of exocytic vesicles, smooth muscle actin-positive stress fibers as well as specialized adhesion complexes resulting in a contractile phenotype [7–9]. Consequently, myofibroblasts secrete high levels of pro-inflammatory and pro-fibrotic paracrine factors as well as ECM proteins, resulting in a disproportionate increase in ECM quantity and changes in ECM quality [10]. Ultimately, cardiac fibrosis eventually leads to deleterious effects on both myocardial contractility and electrophysiology.

This study utilized living myocardial slices (LMS) as a model to simulate cardiac injury and explore potential therapeutic targets by examining novel mechanistic pathways. LMS are highly viable, ultrathin (300 μm) sections of the left ventricular myocardium that retain the native multicellularity, structure and physiology of the heart. They are a platform of intermediate complexity, allowing the modulation of the myocardium at the macroscopic level using, for example, chemical or biological compounds, mechanical load or cryoinjury, while studying cellular responses within a native cardiac environment [11–16]. LMS have been produced from a number of small and large mammals, including mice, rats, rabbits, dogs, pigs and human tissue biopsies or explanted hearts [14,17–19]. Multiple slices can be generated from each heart or biopsy, producing multiple samples for experimentation and thus reducing the number of animals required for studies.

Dixon et al. [20] were the first to propose ferroptosis as a new pattern of iron-dependent programmed cell death that differs from other forms of regulated cell death mechanisms in several aspects, including morphology, biochemistry, and immune status [21]. Ferroptosis is a form of programmed cell death that involves iron-dependent lipid peroxidation. This process directly damages phospholipids and also serves as a cell death signal, triggering programmed cell death [22]. Several recent studies have indicated a potential involvement of ferroptosis in the development and progression of fibrotic diseases. To illustrate, one study demonstrated the effectiveness of ferrostatin-1 (Fer-1), an inhibitor of ferroptosis that works by impeding lipid peroxidation, in reducing renal fibrosis in an animal model of chronic kidney disease [23]. Another study showed that high dietary iron predisposes mice to liver fibrosis, which could be reversed through treatment with Fer-1, suggesting that ferroptosis may underlie the development of hepatic fibrosis [24]. Similarly, in pulmonary fibrotic diseases like idiopathic pulmonary fibrosis, an analysis of human tissue data using bioinformatics techniques provided support for the significant correlation between the expression of ferroptosis-related genes and the progression of the disease [25–27].

Although the precise role of ferroptosis in heart fibrosis is an active area of investigation, various studies have indicated its occurrence in different conditions associated with fibrosis formation, including myocardial infarction, ischemia-reperfusion injury, and heart failure. For instance, in a mouse model of myocardial infarction, the protein glutathione peroxidase 4 (GPX4), which plays a protective role against ferroptosis by targeting lipid peroxides, is found to be downregulated during the early and middle stages of the disease. Consequently, there is an accumulation of lipid peroxides, leading to the initiation of ferroptosis [28]. Furthermore, inhibiting ferroptosis has been shown to protect the mammalian myocardium against ischemia-reperfusion injury through the modulation of the VDAC1 and NRF2 signalling pathways [29,30]. In rats with heart failure with preserved ejection fraction (HFpEF), the administration of an SGLT2 inhibitor not only preserved cardiac function but also suppressed ferroptosis [31]. This finding indicates that ferroptosis may have a significant role to play in advancing our understanding of HFpEF. Therefore, understanding the mechanisms of ferroptosis in the context of cardiovascular diseases can provide valuable insights into disease progression. This knowledge can pave the way for the development of innovative treatment approaches, leading to improved patient care and outcomes.

In this study, we used the *ex vivo* model of LMS to investigate cardiac tissue response to injury using contractile, structural, and translational multi-OMIC analysis. Our findings demonstrated the activation of ferroptosis in this model. Remarkably, therapeutic inhibition of ferroptosis led to notable improvements in structural and molecular parameters during both the acute and chronic phases of injury. These beneficial effects may be attributed to a fibroblast-mediated mode of action, likely through the involvement of metallothionein activity.

## 2 Methods

### 2.1 Animal experiments

The heart samples were obtained from 8- to 12-week-old male Sprague Dawley rats. All animal experiments conducted in this study were registered and approved under §4 Tierschutzgesetz by the LAVES (Niedersächsisches Landesamt für Verbraucherschutz und Lebensmittelsicherheit) of Lower Saxony, with the registration number 2017/117. The animals were housed in the central animal facility of the Medical School Hanover (MHH/ZTL), where specific pathogenic-free conditions were maintained. They had *ad libitum* access to food and water and were housed under controlled humidity, temperature, and lighting conditions.

For heart retrieval, rats were anesthetized with 5 % isoflurane and 2 L/min O_2_ and sacrificed by cervical dislocation. Then, the mediastinum was exposed and the heart was retrieved and washed in heparinized (2.5 IU/mL) modified Tyrode's solution (see ‘Slice preparation and culture conditions’ below): first in warm (37 °C) and then in cold (4 °C) Tyrode's solution, in which they were stored on ice until slicing.

### 2.2 Human tissue acquisition

Human myocardial tissue specimens were obtained by the Clinic for Cardiac, Thoracic, Transplant and Vascular Surgery (HTTG) at the Hannover Medical School (MHH), Hannover, Germany. The study was conducted in accordance with ethical standards and guidelines outlined in the 1964 Declaration of Helsinki and its subsequent amendments. Patients enrolled in the study provided informed consent for the scientific use of the explanted tissue.

### 2.3 Slice preparation and culture conditions

The preparation [32] and culture [11,19] of myocardial slices have been described before. Briefly, modified Tyrode's solution was freshly prepared for tissue storage and processing, containing 30 mM 2,3-butanedione monoxime (BDM),10 mM D-glucose,10 mM HEPES,12 mM KCl,140 mM NaCl, 1 mM MgCl_2_, 0.9 mM CaCl_2_ and 0.33 mM sodium phosphate monobasic dehydrate. Left ventricular tissue blocks were prepared and sliced into 300 μm thick sections using high-precision vibrating microtomes (Model 7000SMZ-2, Campden Instruments LTD), fitted with a temperature-controlled tissue bath filled with 4 °C modified Tyrode's solution. Subsequently, the sections were trimmed into 7 × 7 mm cuts using a razor blade and attached to 3D printed plastic rings using surgical histoacryl (B. Braun, #1050052), then transferred to MyoDish 1 Tissue Culture System (InVitroSys), which are specifically designed cultivating chambers for *ex vivo* culture, as described by Fischer et al. [19]. LMS were cultured in Medium 199 (Sigma-Aldrich, #M4530) supplemented with 1:1000 ITS liquid media supplement (Sigma-Aldrich, #I3146) and 3 % penicillin-streptomycin (Gibco, #15140). In the chambers, LMS were mechanically stretched to a sarcomere length of 2.1 μm, achieved by 12.6 % stretch in rat LMS [14] and 15 % in human LMS [11]. In addition, LMS were electrically stimulated at 0.2 Hz, 20–30 mA, 3 ms. LMS contraction was monitored and recorded throughout the time in culture for further analysis. The functional analysis was done with LabChart software (ADInstruments).

### 2.4 Treatment of LMS

Small molecular compounds, such as ferrostatin-1 and dexrazoxane were solubilized in DMSO. The treatment was subsequently introduced directly into the medium (1:1000) following the preparation of LMS and was homogenously mixed by pipetting up and down. The treatment duration spanned 1 day for rat LMS and 3 days for human LMS. In the latter scenario, a medium replacement procedure was conducted 36 h after the start of the experiment. This was done using fresh Medium 199 supplemented with the desired concentration of the compound. To apply cryoinjury on LMS, a metal probe was cooled down by liquid nitrogen and then pressed against the tissue. In contrast, for the establishment of a non-injury based fibrotic model, the LMS were incubated with 50 ng/mL TGF-β1 (R&D Systems, #7754-BH-025).

### 2.5 Immunohistochemical staining and microscopy

#### 2.5.1 LIVE-DEAD staining

LIVE/DEAD™ Viability/Cytotoxicity Kit, for mammalian cells (Invitrogen, #L3224) was used. Calcein AM and ethidium homodimer-1 were diluted in Medium 199 (Sigma Aldrich, M4530) to the final concentrations of 2 μM and 4 μM, respectively. LMS were incubated in the staining mixture for 5 min at 37 °C, while stretched and electrically stimulated, then moved to cold Tyrode's solution and stored on ice until imaging, which was performed within 30 min.

#### 2.5.2 Immunofluorescence staining

After the culture period, the plastic rings were carefully removed from LMS using a razor blade. Subsequently, the LMS was washed with phosphate-buffered solution (PBS) and fixed in a 4 % paraformaldehyde (PFA) solution for 15 min at room temperature (RT). LMS staining and imaging were performed as previously described [11]. Briefly, LMS were permeabilized in 1 % Triton (Roth 9002) diluted directly in blocking solution (composed of 10 % fetal bovine serum (Gibco 078 K), 10 % horse serum (Gibco 16,050,122) and 5 % bovine serum albumin (Gerbu 1063) in PBS) and incubated overnight at 4 °C. The primary and secondary antibodies were diluted in PBS. The samples were incubated overnight at 4 °C with the primary antibodies and for 2 h in RT with the secondary antibodies. Used antibodies and dilutions are stated in Table 1.

Image acquisition was performed using a Leica Inverted 3 confocal microscope. *Z*-stack images were acquired and processed using Fiji ImageJ [33].

#### 2.5.3 Quantification of cell death

Click-iT™ Plus TUNEL-Assay-Kit Alexa Fluor 488 (Thermo Fischer, C10617) was used. PFA fixated samples were embedded in paraffin, cut into 3 μm sections using a microtome (Leica), and rehydrated using xylol-isopropanol- dH2O. After rinsing the samples with PBS and dH2O, 100 μL TdT Reaction Buffer (Component A) was added to each slide and incubated for 10 min at 37 °C. Subsequently, the solution as removed and 50 μL of TdT reaction mixture was added and each sample was incubated for 1 h at 37 °C. The solution was rinsed off with dH2O and PBS for 3 min, before 50 μL Click-iT Plus TUNEL reaction cocktail was added and the samples were incubated for 30 min at 37 °C in the dark. The last steps were all performed with as little light as possible. First, the samples were again washed with PBS, before a background staining with Phalloidin (Sigma, P1951) was performed. For this, the solution was diluted 1: 100 in PBS and the samples were incubated 1 h at 4 °C. Lastly, all nuclei were stained with Hoechst 33342 (Invitrogen, H3570). Here, the stain was diluted 1: 1000 and the samples were incubated for 10 min at RT. After the dye was washed off with PBS three times for 5 min, the stained samples were covered with one drop of mounting medium ProLong™Gold antifade reagent (Invitrogen, P36930), before a cover slip was placed over them and the mounting medium was dried at RT for 24 h. Image acquisition was performed using a fluorescence microscope (Keyence, BZ-X810) and processed using Fiji ImageJ. To calculate the amount of apoptotic cells, first the entirety of all cells and TUNEL-positive cells was determined, before a ratio was calculated. At the department of clinical chemistry at the medical school of Hannover, 200 μL of supernatant was tested for extracellular lactate dehydrogenase (LDH) (Cobas C701, Module ISE, Roche). Results were normalized to volume of respective LMS.

### 2.6 RNA extraction and real-time qPCR (RT-qPCR)

RNA was extracted from 200 μL supernatant using the miRNeasy Serum/Plasma Advanced Kit (Qiagen, #217204). Cells were collected in QIAzol Lysis Reagent (Qiagen, #79306) and incubated for 5 min, then stored in −80 °C for further processing. Whole LMS were homogenized in QIAzol for 40 s at 5500 RPM using Precellys 24 homogenizer (Bertin Technologies) and stored in −80 °C for further processing. RNA extraction from LMS/cell lysate was performed using the precipitation method or RNeasy Mini Kit (Qiagen, #217004) according to the manufacturer's instructions. Reverse transcription of mRNA was performed with iScript Select cDNA Synthesis Kit (Bio-Rad, #170–8897) using oligo dT primers. cDNA was diluted 1:4 with dH2O prior to RT-qPCR with iQ SYBR Green Supermix (Bio-Rad, #1708882). A mixture of forward and reverse primer pairs (10 μM, Eurofins) was used for quantifying mRNA. To quantify miRNA, RNA was reverse-transcribed using the miRNA TaqMan MicroRNA Reverse Transcription Kit (Applied Bio-systems, #4366597). cDNA was diluted 1:3 with dH2O before qPCR with Absolute Blue qPCR Mix (Abgene, #AB-4136/B) and the specific Taqman probes (ThermoFisher, #4427975). Runs were performed in ViiA7 (Applied Biosystems) or QuantStudio 7 Flex (Applied Biosystems). RT-qPCR data were analyzed using the ΔΔ-CT method and presented as fold change compared to the reference genes: GAPDH for mRNA and cel-miR-39 for miRNA. Primer sequences are found in Table 2.

### 2.7 RNA sequencing

250 ng of total RNA per sample were utilized as input for mRNA enrichment procedure with ‘NEBNext® Poly(A) mRNA Magnetic Isolation Module’ (E7490L; New England Biolabs) followed by stranded cDNA library generation using ‘NEBNext® Ultra II Directional RNA Library Prep Kit for Illumina’ (E7760L; New England Biolabs). All steps were performed as recommended in user manualE7760 (Version 1.0_02–2017; NEB) except that all reactions were downscaled to 2/3 of initial volumes. cDNA libraries were barcoded by dual indexing approach, using ‘NEBNext Multiplex Oligos for Illumina – 96 Unique Dual Index Primer Pairs’ (6440S; New England Biolabs). All generated cDNA libraries were amplified with 9 cycles of final PCR. One additional purification step was introduced at the end of the standard procedure, using 1.2× ‘Agencourt® AMPure® XP Beads’ (#A63881; Beckman Coulter, Inc.). Fragment length distribution of individual libraries was monitored using ‘Bioanalyzer High Sensitivity DNA Assay’ (5067–4626; Agilent Technologies). Quantification of libraries was performed by use of the ‘Qubit® dsDNA HS Assay Kit’ (Q32854; ThermoFisher Scientific).

Equal molar amounts of individually barcoded libraries were pooled for a common sequencing run in which each analyzed library constituted around 6.3 % of overall flowcell / run capacity. The library pool was denatured with NaOH and was finally diluted to 1.8pM according to the Denature and Dilute Libraries Guide (Document # 15048776 v02; Illumina). 1.3 m-L of the denatured pool was loaded on an Illumina NextSeq 550 sequencer using a High Output Flowcell (400 M cluster) for single reads (20,024,906; Illumina). Sequencing was performed with the following settings: Sequence reads 1 and 2 with 38 bases each; Index reads 1 and 2 with 8 bases each.

Raw data processing was conducted by use of nfcore/rnaseq (version 1.4.2) which is a bioinformatics best-practice analysis pipeline used for RNA sequencing data at the National Genomics Infrastructure at SciLifeLab Stockholm, Sweden. The pipeline uses Nextflow, a bioinformatics workflow tool. It pre-processes raw data from FastQ inputs, aligns the reads and performs extensive quality-control on the results. The genome reference and annotation data were taken from GENCODE.org (*Homo sapiens*: GRCh38.p13; release 34).

Normalization and differential expression analysis was performed on the internal Galaxy (version 20.05) instance of the RCU Genomics, Hannover Medical School, Germany with DESeq2 (Galaxy Tool Version 2.11.40.6). The deregulated (p-adj < 0.05 and log_2_(FC) > |0.5|) genes were analyzed with ORA WEB-based GEne SeT AnaLysis Toolkit (Web-Gestalt) [34] using the GO Biological Process, GO Molecular Function, GO cellular component, KEGG, Reactome and Wikipathway databases (*Homo sapiens*), with default parameters except “FDR 0.05”. Selected significantly enriched database entries were plotted with GraphPad Prism (version 9.3.1).

### 2.8 Protein extraction, western blot and proteomics

Whole LMS were homogenized in a mixture of cell lysis buffer (Cell Signalling Technology, #9803) and pefabloc SC (Sigma-Aldrich, #76307) for 20 s at 5000 RPM using Precellys24.

For western blot, samples were pipetted onto polyacrylamide gels for electrophoretic band separation and then transferred to an Immun-Blot PVDF Membrane (Biorad, #1620177). Blocking of membranes was performed by incubation for 1 h in 5 % milk solution. The membranes were incubated with the primary antibody overnight at 4 °C. After washing, the membranes were incubated with HRP-linked secondary antibody for 1 h at RT, ultimately being washed again and visualized *via* the enhanced chemiluminescence (ECL) reagent (Biorad, #1705061). Antibodies used for the detection of protein targets are stated in Table 1.

For proteomics analysis, proteins were separated on pre-casted SDS gels (BioRad) and stained with Coomassie solution (Thermo Fisher #20279). Gel lanes were sliced into 4 pieces and after destaining proteins were in-gel digested with trypsin overnight. Peptides were analyzed by Orbitrap-MS and standard conditions with DDA MS data were searched against *rattus norvegicus* entries of Uniprot DB (unreviewed version UP000002494, containing 31,571 entries). Filtering was done that three values have to be quantified for a protein group at least in one sample group. Missing values were imputated with a downshift of 1.8 (log to basis of 2) and a width of 0.3. 2178 protein groups were used for bioinformatic analysis. Mean ratios of all three groups (control, CI *peri*, CI remote) were calculated and ratios were determined. Samples were tested using multiple sample testing with ANOVA (Sidak *post hoc* test) and subsequent adjustment of multiple comparisons (Storey-Tibshirani procedure). The deregulated (q-adj. <0.05 and LQF intensity difference x > |0.5|) proteins were analyzed with ORA WEB-based GEne SeT AnaLysis Toolkit (WebGestalt) [34] using the GO Biological Process, GO Molecular Function, GO cellular component, KEGG and Reactome pathways databases (*Rattus norvegicus*), with default parameters except “FDR 0.05”. Selected significantly enriched database entries were plotted with GraphPad Prism (version 9.3.1).

### 2.9 Human cardiac fibroblasts (HCF) culture

Cryopreserved HCFs of several donors (Promocell, #C-12375) were thawed and transferred into fibroblast growth medium (FGM-3), which consists of Fibroblast Basal Medium 3 (Promocell, #C-23230) supplemented with 1 ng/mL Basic Fibroblast Growth Factor (bFGF), 5 μg/mL Insulin (Promocell, #C-39350), 1 % Penicillin–Streptomycin (P/S, Gibco, #15140122) and 10 % Fetal Bovine Serum (FBS, Gibco, #10270106). FGM-3 was exchanged 24 h after thawing and/or 96 h after passaging. Cells were split every 7 days using Trypsin/EDTA (Invitrogen, #25300096).

### 2.10 In vitro migration assay

HCFs were seeded into 0.1 % gelatine-coated 96-well plates at 30,000 cells per well. After 24–48 h of incubation, when confluence reached 90–100 %, Hoechst 33342 (Thermo Fisher, #H3570) staining was added for 15 min at 37 °C, then scratches were introduced manually using a small pipette tip. After washing in PBS, fresh medium premixed with the drug or vehicle compounds was added and the plates were imaged every 2 h using Cytation-1 Cell Imaging Multimode Reader (BioTek) and BioSpa 8 Automated Incubator (BioTek). The generated images were analyzed using Fiji Image-J software.

In parallel to the migration assay, the cytotoxicity was analyzed using CellTox™ Green Cytotoxicity Assay (Promega G8741). CellTox™ Green Dye (1:1000) was added shortly before imaging. The readout for the CellTox™ assay is an increased fluorescence intensity in case of impaired membrane integrity.

### 2.11 Reactive oxygen species (ROS) production assay

ROS production was assessed using the DCFDA / H2DCFDA - Cellular ROS Assay Kit (Abcam, ab113851). Briefly, cells were seeded in 96-well-plate until complete confluence and then stained with DCFDA dye (1:1000 diluted in 1× assay buffer) for 45 min at 37 °C. After removing the dye and washing the cells with PBS, the treatments were added (in 1× assay buffer) and the fluorescence intensity was measured *via* Cytation 1 (BioTek) every 20 min for the duration of 6 h. All treatments were added with and without H_2_O_2_.

### 2.12 NF-κB signalling assay

A Luciferase Reporter Assay in HEK293 cells was used to determine the activity of inflammatory NF-κB signalling. In a 48-well format, 175,000 HEK293FT cells were co-transfected with the pSGNluc plasmid, a β-galactosidase control plasmid (Promega, United States). The pSGNluc plasmid contains multiple NF-κB binding sites and therefore, enables evaluating NF-κB activity *via* detection of Luciferase activity. After transfection, medium was changed to serum-free medium with and without 300 ng/mL Poly I:C and supplemented with DMSO, Fer-1 or DXZ treatment. Then cells were incubated with these treatments for 24 h at 37 °C and 5 % CO2. Luminescence was detected using a Synergy HT Multi-Detection Microplate Reader (BioTek, United States) and in the end, Luciferase activity was normalized to β-galactosidase activity.

### 2.13 Ferroptosis sensitization assays

HCFs were seeded into 96-well plates at a density of 30,000 cells per well. After a 24-h incubation period, transfection with MT1F siRNA or control siRNA (Santa Cruz, sc-93,381) was performed. The siRNA was diluted in Opti-MEM™ I Reduced Serum Medium (Gibco, #31985070) to a final concentration of 10 μM. In a separate preparation, Lipofectamine RNAiMAX (Invitrogen, #13778150) was diluted 1:125 in Opti-MEM™ I Reduced Serum Medium. The two mixtures were combined after a 5-min incubation at room temperature and further incubated for an additional 20 min at room temperature. HCFs were then exposed to the transfection mixture for 6 h at 37 °C with 5 % CO2, after which the medium was replaced with fresh culture medium. After 48 h, HCFs were treated with increasing concentrations of RSL-3 (Sigma, SML2234), and cell viability was assessed using two methods. Firstly, the WST-1 assay was performed using the Cell Proliferation Reagent WST-1 solution (Roche, catalog no.: 05015944001). In brief, the WST-1 reagent was diluted 1:10 in fresh culture medium. After removing the existing medium from the cells, 100 μL of the freshly prepared medium containing the WST-1 reagent was added to each well. The cells were then incubated at 37 °C for 4 h. Absorbance measurements were taken at 450 nm and 630 nm using the Cytation-1 Cell Imaging Multimode Reader (BioTek). For data analysis, absorbance values were first normalized to the average of the “blank” wells, followed by the subtraction of the 630 nm value from the 450 nm value for each well. Secondly, cell death quantification was performed using the CellTox™ Green Cytotoxicity Assay (Promega, G8741). Hoechst 33342 staining (Thermo Fisher, H3570) was applied 1:1000 for 15 min at 37 °C immediately before RSL-3 treatment to determine the total cell count. CellTox™ Green Dye, diluted 1:1000, was added just prior to imaging. Cell counting was conducted using the Cytation-1 Cell Imaging Multimode Reader. For data analysis, the number of dead cells was divided by the total number of cells in each well.

### 2.14 Statistical analysis

All statistical analyses were carried out employing GraphPad Prism (versions 8 or 9). For the comparison of two groups, an unpaired two-tailed Student's *t*-test was applied. In cases where three or more groups were compared, a one-way ANOVA was employed. In instances involving two distinct variables, a two-way ANOVA was executed, followed by post-hoc tests using Tukey's or Dunnett's methods. All numerical values are presented as the mean ± SEM, unless explicitly specified otherwise. Throughout all experiments, statistical significance was defined as a *p*-value of equal to or less than 0.05 (*: *p* ≤ 0.05, **: *p* ≤ 0.01, ***: *p* ≤ 0.001, ****: *p* ≤ 0.0001).

## 3 Results

### 3.1 Cryoinjury impairs cardiac function in adult rat LMS

**Fig. 1:**
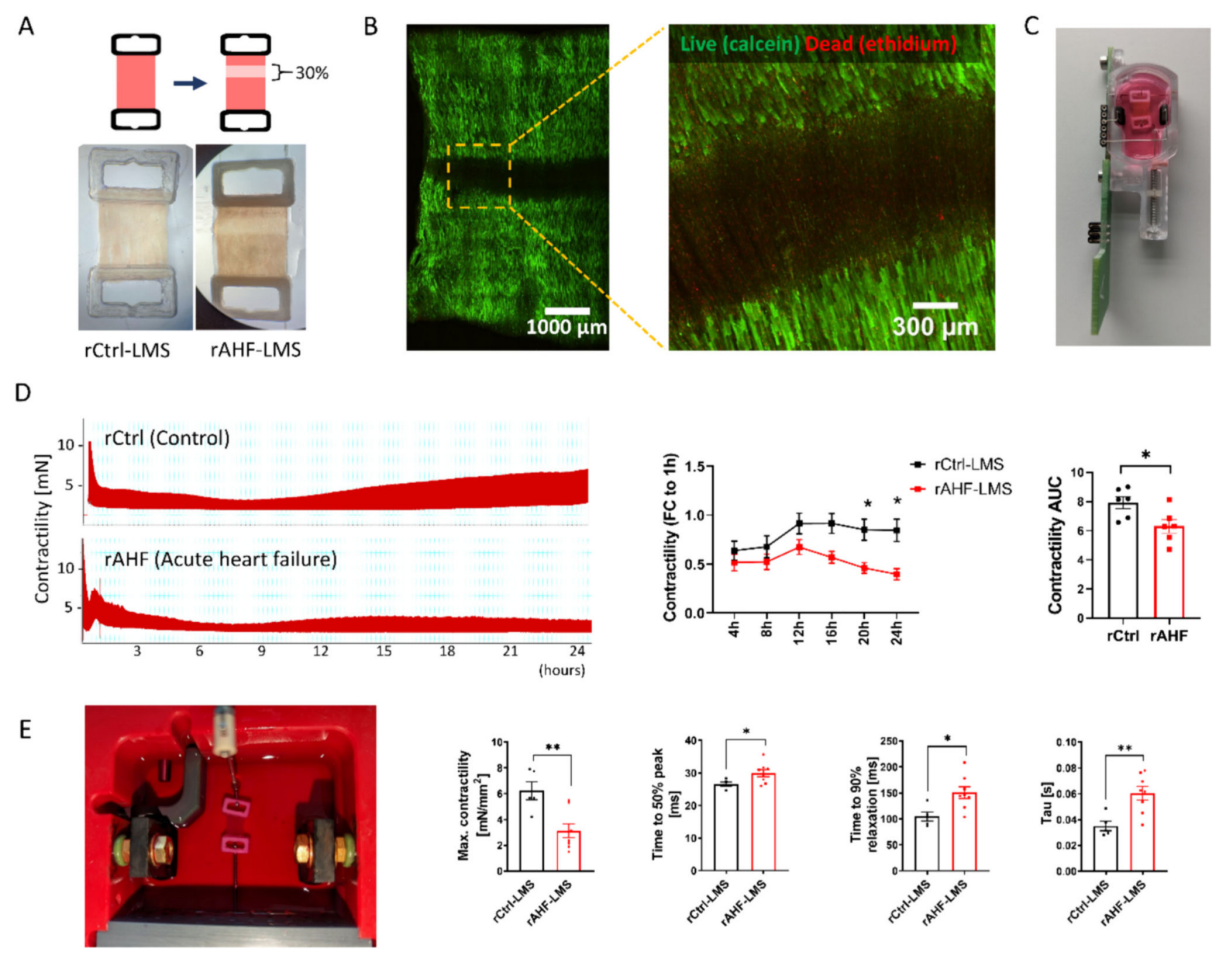
Cryoinjury in healthy rat LMS induces cardiac dysfunction. (A) Freshly cut LMS were trimmed based on fiber orientation and attached to plastic rings. Left – intact healthy LMS (rCtrl-LMS). Right – cryioinjured LMS (rAHF-LMS). (B) Representative image of live/dead staining of rAHF-LMS imaged using wide-field microscopy. (C) View of a LMS in biomimetic cultivation chamber (BMCC - InVitroSys). (D) Left – Representation of continuous contractility recording from rCtrl- and rAHF-LMS over 24 h. Middle - Time course of peak amplitude (contractility) during culture in BMCC, shown as fold change to 1 h values (*p < 0.05; two-way ANOVA; *n* = 6). Right – area under curve (AUC) of contractility over 24 h (*p < 0.05; Student's *t-*test; n = 6). (E) Force measurement of LMS in the force transducer and quantification of contractile parameters: Maximal contractility, Time to 50 % peak, Time to 90 % relaxation and Tau (τ). (*p < 0.05; Student's t-test; n_rCtrl-LMS_ = 5, n_rAHF-LMS_ = 8). Data are displayed as mean ± SEM.

Upon cardiac injury, the adult mammalian heart suffers from cardiomyocyte deficiency and hence a lack of functional recovery of the infarcted or failing heart. Individuals who experience myocardial infarction lose on average 25–30 % of their left ventricular area [35,36]. To mimic this clinical scenario, we subjected freshly prepared adult rat LMS to cryoinjury comprising 30 % of their total area with the aim to model post-MI changes *ex vivo*. To apply cryoinjury on LMS, a metal probe was cooled down by liquid nitrogen and then pressed against the tissue (Fig. 1A; Video 1). Assessment of cellular viability by LIVE-DEAD staining revealed complete cellular death in the cryoinjured area, while adjacent cells were found to be highly viable (Fig. 1B). Both the control LMS (rCtrl-LMS) and injured LMS (rAHF-LMS, representing rat acute heart failure LMS) were cultivated under the same conditions in biomimetic cultivating chambers (BMCC). Throughout the *ex vivo* culture, LMS were exposed to electrical pacing to induce contractions, while their contractile function was consistently monitored (Fig. 1C). Evaluation of the contractions recorded in the BMCC revealed diminished contractility in rAHF-LMS compared to the control group, coinciding with the loss of CMs. These changes were most pronounced after 20 h in culture (Fig. 1D). Other contractile parameters remained unchanged (Supplementary fig. 1). At the end of cultivation (24 h), LMS were mounted on a force transducer (Harvard Apparatus) for force measurements. Unlike the measurement performed in BMCCs, the force transducer allows for assessing the maximal contractility, which is achieved by applying increasing stretch on LMS according to the Frank-Starling law. In this analysis, we found a significant reduction in the maximal contractility of rAHF-LMS, in addition to prolonged time to 50 % peak (TP50%), relaxation time (TR90%), and Tau (Fig. 1E). Taken together, these findings suggest a decline in the contractile function of LMS after cryoinjury, indicating that rAHF-LMS model replicates the functional impairment observed in patients experiencing acute heart failure following myocardial infarction.

### 3.2 Cryoinjured LMS mimic features of cardiac remodelling

**Fig. 2:**
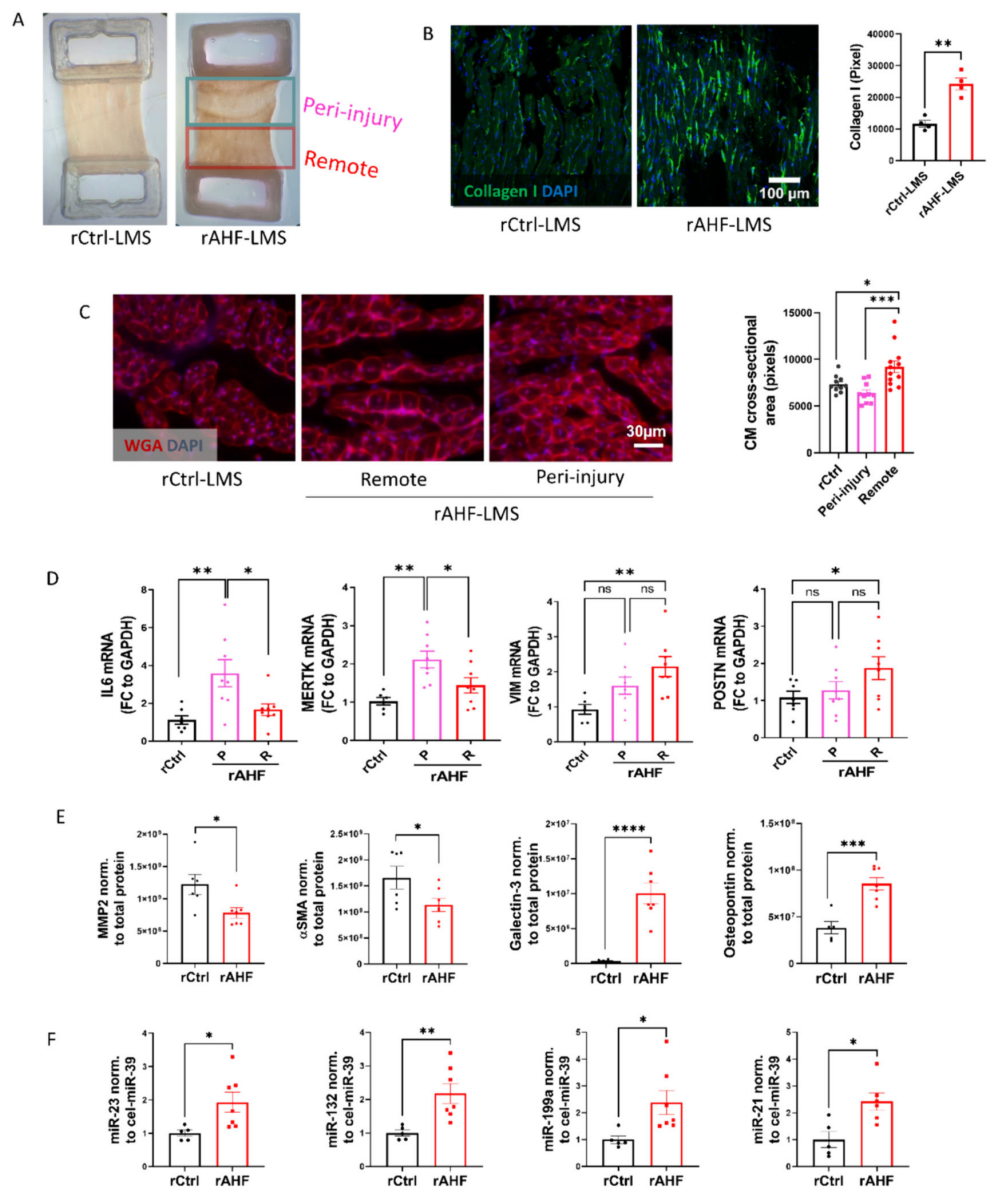
Cryoinjury of LMS induces remodelling in the region adjacent to the injury. (A) rCtrl- and rAHF-LMS after 24 h in culture. rAHF were split inpto peri-injury (cryoinjury +1 mm margins) and remote regions. (B) Representative images and quantification of collagen I immunostaining in rCtrl-LMS and the remote regions of rAHF-LMS (**p < 0.01; Student's t-test, *n* = 4). (C) Cross sections of LMS were stained with wheat germ agglutinin (WGA) to demarcate cell boundaries. Right - quantitative analysis of cardiomyocyte cross-sectional area (*p < 0.05, ***p < 0.001; one-way ANOVA; n_rCtrl_ = 9, n_peri-injury_ = 10, n_remote_ = 12). (D) RT-qPCR quantification of gene expression in LMS. P = peri-injury, R = remote (*p < 0.05, **p < 0.01; one-way ANOVA; n_rCtrl_ = 6, n_rAHF_ = 8). (E) Western blot analysis of secreted proteins into the supernatant, normalized to total protein measured *via* Ponceau S staining (**p < 0.01, ***p < 0.001; Student's t-test; n_rCtrl_ = 6, n_rAHF_ = 7). (F) RT-qPCR quantification of miRNAs secreted to the supernatant, normalized to cel-miR-39 (*p < 0.05, **p < 0.01; Student's t-test; n_rCtrl_ = 5, n_rAHF_ = 7). Data are displayed as mean ± SEM.

To explore the potential of our model to replicate the remodelling process that ensues after cardiac injury *in vivo*, we focused on alterations in morphology, gene expression and paracrine behavior. To gain a deeper understanding of the spatial effects of injury on cardiac tissue, we decided to divide rAHF-LMS into two parts: peri-injury (rAHF-PI) – encompassing the cryoinjured region with 1 mm margins, and remote (rAHF-R) – consisting of the remaining portion of the slice (Fig. 2A). The cross-sectional area of CMs (CM-CSA) was quantified using wheat germ agglutinin (WGA) staining, demonstrating CM hypertrophy in the remote regions with an average increase of 25 % in CM-CSA. Conversely, CM-CSA in the outskirts of the peri-injury regions remained unaltered (Fig. 2C).

To assess the presence of cardiac fibrosis in our model, we examined the deposition of extracellular matrix in the different regions of rAHF-LMS. Collagen I staining revealed stronger expression within the interstitial space of the remote regions of rAHF-LMS, indicating the activation of cardiac myofibroblasts in response to the injury (Fig. 2B). This observation was further supported by the upregulation of fibroblast activation-associated genes, such as periostin and vimentin, in the remote regions of rAHF-LMS, while these genes showed no significant changes in the peri-injury regions. In contrast, genes associated with inflammatory pathways, such as IL6 and MerTK, displayed stronger upregulation in the peri-injury regions (Fig. 2D). These findings not only indicate that our model effectively replicates various molecular processes observed in *in vivo* models of cardiac injury but also demonstrates comparable spatial distribution patterns. We next conducted quantification of protein secretion associated with fibroblast activation in the supernatant (Fig. 2E; supplementary fig. 2). Galectin-3 and osteopontin were found to be significantly elevated in rAHF-LMS. These proteins have been previously proposed as potential markers of fibroblast activation and prognostic factors in heart failure patients [37,38]. MMP2 and αSMA, on the other hand, were found to be less abundant in the supernatant of rAHF-LMS. This trend was observed in previous studies as well, suggesting that these proteins may undergo redirection into intracellular activity following cardiac injury [39–41]. Additionally, we quantified microRNAs (miRNAs) associated with cardiac disease and repair in the supernatant. miRNAs are known to be secreted by various cell types, making them promising candidates for biomarkers and companion diagnostics. We observed enhanced abundance of miR-199a, a known biomarker of acute myocardial infarction, as well as miR-23, miR-132 and miR-21, all of which known to play critical roles in CM biology, cardiac fibroblast activation and ECM deposition.

Taken together, these findings demonstrate that rAHF-LMS possess key characteristics of myocardial infarction and cardiac injury, including inflammation, hypertrophy, and fibrosis. The resemblance to *in vivo* settings highlights the translational significance of this model for drug discovery and testing purposes. Furthermore, rAHF-LMS serves as an excellent platform for studying secretory pathways and potentially discovering novel biomarkers for the diagnosis and assessment of cardiac injury.

### 3.3 Identification of key molecular networks in cardiac injury

Comparative quantitative proteomic analysis not only offers the global expression pattern of thousands of proteins but also unveils the potential pathways regulated in the LMS under certain conditions, which could be targeted for therapeutic purposes [42].

**Fig. 3:**
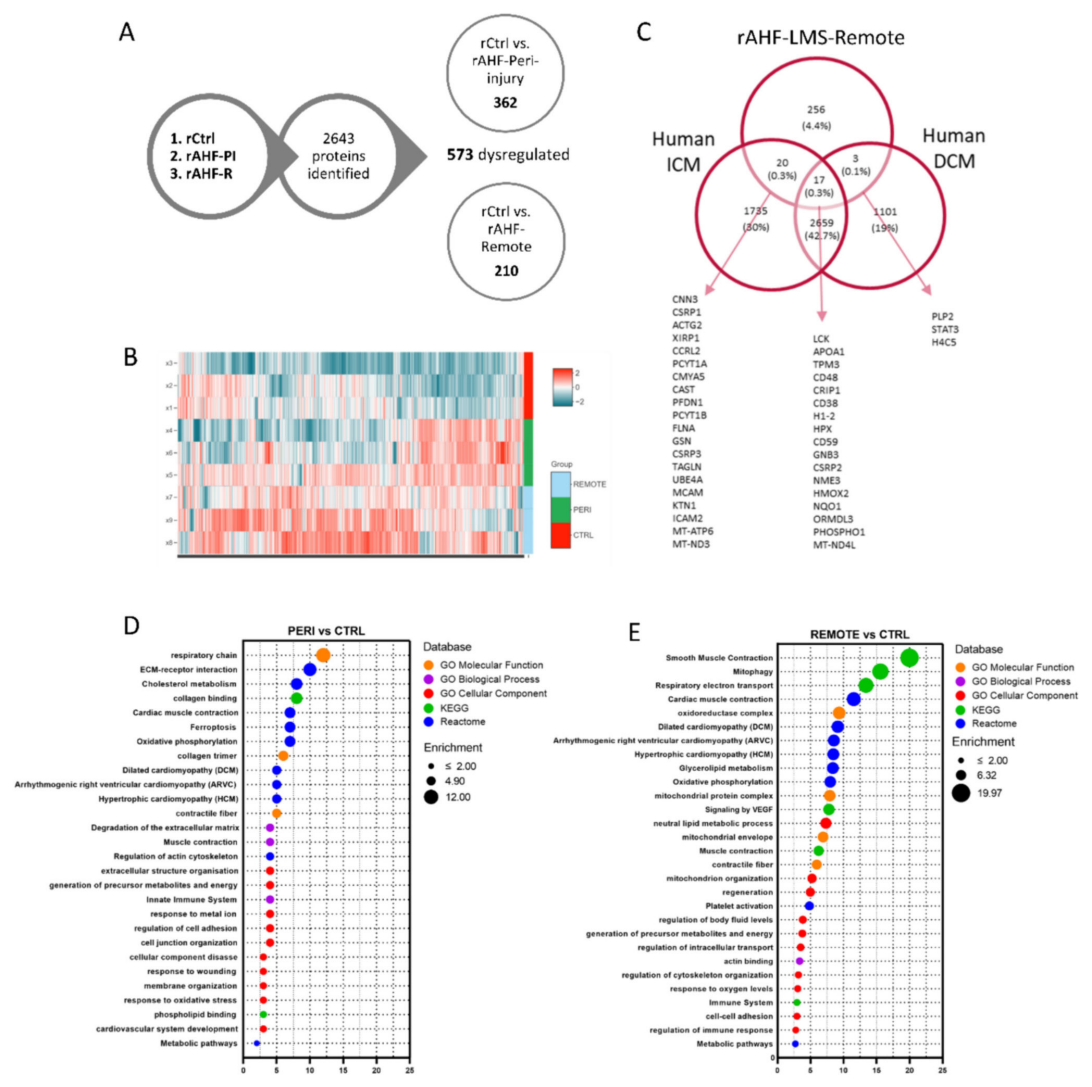
Mass spectrometry-based proteomics analysis of cryoinjured LMS. (A) Diagram of proteomics analysis. 3 groups were analyzed: rCtrl-LMS, rAHF-remote (rAHF-R) and rAHF-peri-injury (rAHF-PI) (p-ajd < 0.05; |Log_2_FC| > 0.5; *n* = 3). (B) Heatmap illustrating protein levels. Color scaling shows the levels for given proteins, red - highest, blue – lowest. (C) Venn diagram showing the overlap between DAPs in our proteomics dataset and DEGs found in a previously published RNA seq dataset from ICM and DCM patients (GSE116250). (D + E) Functional enrichment analysis of proteomics data. Selected clusters from different databases are included. X-axis: Normalized enrichment score rAHF-R/rAHF-P *vs*. rCtrl. Bubble size illustrates the ratio between the proteins connected to a process and the overall query size. Color of the bubble correlates with the database source. FDR < 0.05. (For interpretation of the references to color in this figure legend, the reader is referred to the web version of this article.)

We performed high-throughput proteomics analysis to compare the protein abundancy in three groups: control rat LMS (rCtrl), acute heart failure – peri-injury (rAHF-PI) and acute heart failure – remote (rAHF-R). The objective of this comparison was to identify proteins associated with the subacute phase of cardiac injury, with the ultimate goal of pinpointing potential targets for therapeutic intervention. With |log_2_FC| > 0.5 and pajd<0.05, this analysis identified 2643 proteins, 573 of which were found to be differentially abundant proteins (DAPs): rCtrl *vs*. rAHF-PI – 362 DAPs, rCtrl *vs*. rAHF-R – 210 DAPs (Fig. 3A-B; supplementary fig. 3; supplementary table 1). To gain further insights, we performed bioinformatics analysis and mapped our results to a previously published transcriptomics dataset from patients with dilated and ischemic cardiomyopathy (GSE116250) [43]. Despite the comparison being between rat proteomics and human transcriptomics data, we identified a significant overlap of 40 genes/proteins, providing further support for the translational value of our model (Fig. 3C).

Functional enrichment analysis revealed a significant enrichment of protein sets associated with various forms of cardiomyopathy, including dilated cardiomyopathy (DCM), hypertrophic cardiomyopathy (HCM), and arrhythmogenic right ventricular cardiomyopathy (ARVM), in both rAHF-PI and rAHF-R when compared to rCtrl. Additionally, there was a notable upregulation of protein sets involved in energy production, *e.g*. respiratory chain and oxidative phosphorylation. This suggests that the cardiac slices may be compensating for the injury-induced contractile impairment by enhancing energy-generating pathways. Pathways related to structural changes and tissue remodelling, such as collagen trimming, degradation of the extracellular matrix, extracellular structure organization, and cellular component disassembly, were also enriched in both groups, with a greater prominence observed in rAHF-PI. Interestingly, proteins associated with the activation of the innate immune system and cholesterol metabolism were found to be specifically enriched in rAHF-PI, while not exhibiting significant enrichment in rAHF-R (Fig. 3D-E).

The proteomics analysis revealed an intriguing enrichment of proteins associated with the KEGG pathway ferroptosis. Ferroptosis is a recently discovered form of iron-dependent cell death characterized by iron accumulation and lipid peroxidation. The ferroptosis pathway showed significant enrichment in rAHF-PI, with the presence of key regulatory proteins such as GPX4, TFRC, VDAC2/3, and FTH-1. Although this enrichment was not observed in rAHF-R, other protein clusters related to lipid peroxidation and mitochondrial function were identified, suggesting a potential association with ferroptosis. These clusters included glycerolipid metabolism, neutral lipid metabolic process, mitochondrion organization, and mitochondrial envelope. These findings are promising and indicate that targeting ferroptosis could be a viable strategy for therapeutic intervention in cardiac injury.

### 3.4 Blocking ferroptosis-related pathways demonstrates cardioprotective effects in both acute and chronic models of heart failure

**Fig. 4:**
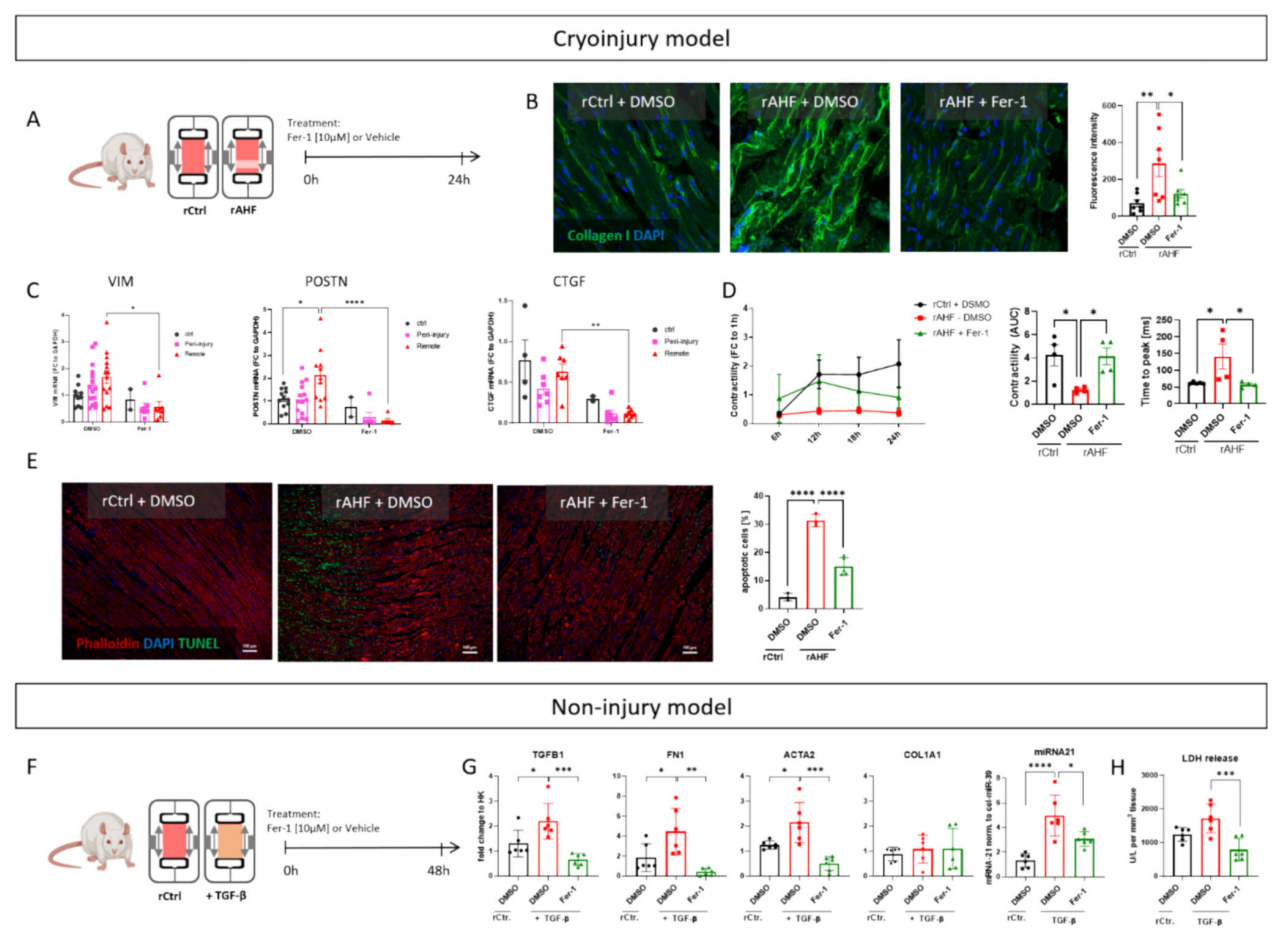
Cardioprotective effects *via* inhibition of ferroptotic pathways in acute heart failure model *ex vivo*. (A) Experimental design of ferroptosis inhibition (*via* Fer-1) in acute heart failure model in adult rat LMS. (B) Representative images and quantification of collagen I immunostaining in rCtrl and rAHF treated with either DMSO (1:1000) or Fer-1 [10 μM] (*p < 0.05, **p < 0.01; one-way ANOVA, *n* = 7). (C) RT-qPCR quantification of gene expression in rCtrl- and rAHF-LMS (*p < 0.05, **p < 0.01, ****p < 0.0001; two-way ANOVA; *n* = 12 for all conditions, except for ctrl + Fer-1 with *n* = 2). (D) Left - time course of peak amplitude (contractility) during culture in BMCC, shown as fold change to 1 h values (n = 4). Middle – AUC of contractility over 24 h (*p < 0.05; one-way ANOVA; n = 4). Right – time to peak analysis (*p < 0.05; one-way ANOVA; n = 4). (E) Representative images and quantification of TUNEL staining in rCtrl and rAHF treated with either DMSO (1:1000) or Fer-1 [10 μM] (****p < 0.0001; one-way ANOVA, n = 3). (F) Experimental design of ferroptosis inhibition (*via* Fer-1) in a non-injury based fibrotic model in adult rat LMS stimulated with 50 ng/μL TGF-β1. (G) RT-qPCR quantification of gene expression in rCtrl- and + TGF-β LMS (*p < 0.05, **p < 0.01, ***p < 0.001, ****p < 0.0001; two-way ANOVA; n = 6). (H) LDH release measured in supernatants collected from rCtrl- and + TGF-β LMS (***p < 0.001; one-way ANOVA; n = 6).

To investigate the role of ferroptosis-related pathways in cardiac injury, we treated intact and injured rat LMS (rCtrl- and rAHF-LMS) with ferrostatin (Fer-1), a chemical inhibitor of ferroptosis that halts lipid peroxidation (Fig. 4A). After 24 h of cultivation, rAHF-LMS treated with Fer-1 demonstrated significantly reduced levels of collagen I compared to those treated with vehicle control, as determined by immunofluorescence staining (Fig. 4B). In line, the expression of genes associated with fibroblast activation, *e.g*. vimentin, periostin and CTGF, were found to be repressed in the remote regions of Fer-1 treated rAHF-LMS compared to their vehicle-treated counterparts (Fig. 4C). Functionally, Fer-1 treatment proved to be effective in improving contractile impairment following injury. We observed a significant increase in the contractility of rAHF-LMS upon treatment with Fer-1. Additionally, the time to peak contraction was reduced, indicating an improvement in the contractile kinetics (Fig. 4D). These functional changes suggest that Fer-1 treatment has a positive impact on the contractile properties of rAHF-LMS, which could be potentially translated into improved ejection fraction and cardiac output in the *in vivo* settings. Parameters related to relaxation were not affected by the treatment (supplementary fig. 4A). Additionally, TUNEL staining was performed to evaluate tissue viability. The results showed reduced cell death in rAHF-LMS treated with Fer-1 compared to the control group (Fig. 4E). To investigate the influence of Fer-1 on cardiac fibroblast activation in a 3D model, we set up a non-injury based model incubating rat LMS with 50 ng/μL TGF-β1 (R&D Systems) for 48 h (Fig. 4F). Here, the expression of genes associated with fibroblast activation, such as TGF β1 (*TGFB1*), fibronectin (*FN1*) and αSMA (*ACTA2*), was found to be increased upon TGF-β1 stimulation and decreased again when Fer-1 was added to the culture simultaneously. The same effect could be observed for the secretion of miR-21, a miRNA associated with fibroblast activation, into the culture medium (Fig. 4G). The expression of collagen 1 (COL1A1), however, was not impaired by either the stimulation with TGF-β1 or treatment with Fer-1. Lastly, the activity of lactate dehydrogenase (LDH) in the supernatant was measured to assess the tissue viability and cytotoxicity of either the TGF-β1 stimulation or the Fer-1 treatment (Fig. 4H). Here, a significant decrease of LDH release could be observed, when LMS were cultured with combination of TGF-β1 and Fer-1.

Overall, our results demonstrate that inhibiting ferroptotic pathways in rAHF-LMS and rat LMS incubated with TGF-β1 exert cardioprotective effects, as evidenced by improvements in cardiac function, reduced fibroblast activation and improved tissue viability.

**Fig. 5:**
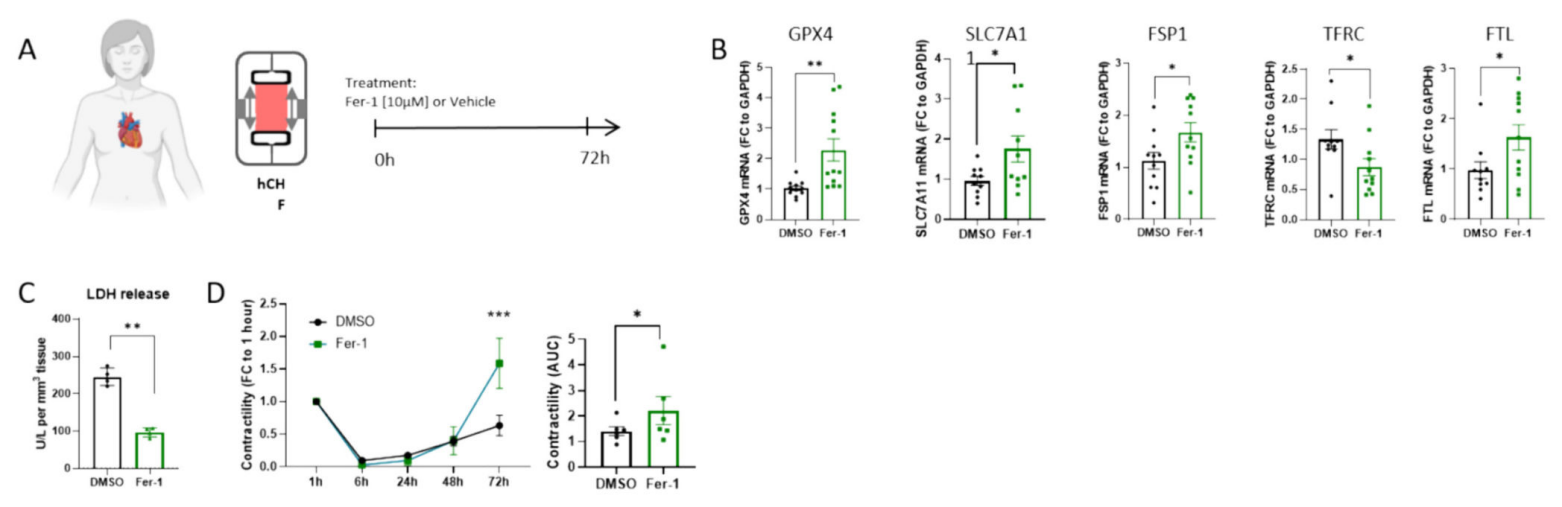
Cardioprotective effects *via* Fer-1 mediated inhibition of ferroptotic pathways in chronic heart failure *ex vivo* model. (A) Experimental design of ferroptosis inhibition (*via* Fer-1) in chronic heart failure model in human LMS. (B) RT-qPCR quantification of ferroptosis-related gene expression in hCHF (*p < 0.05, **p < 0.01; Student's t-test; n_DMSO_ = 11, n_Fer-1_ = 12). (C) LDH release detected in supernatants collected from cultured hCHF LMS (**p < 0.01; one-way ANOVA; n = 4). (D) Left - time course of peak amplitude (contractility) during culture in BMCC, shown as fold change to 1 h values (***p < 0.001; two-way ANOVA; n_DMSO_ = 5, n_Fer-1_ = 6). Right – AUC of contractility over 72 h (*p < 0.05; Student's t-test; n = 6). Data are displayed as mean ± SEM.

While our experiments in rat LMS highlight the significant role of ferroptosis-associated pathways in the acute settings of cardiac injury, heart failure and cardiac fibroblast activation, previous studies have indicated potential involvement in chronic heart failure patients and animal models. To investigate the role of ferroptotic pathways and their inhibition in the context of chronic heart failure, LMS were generated from failing human hearts obtained at the time of heart transplantation (Fig. 5A). Enrolled patients were diagnosed with either dilated cardiomyopathy (DCM) or ischemic cardiomyopathy (ICM) (Supplementary table 2). Treating the human heart failure LMS (hCHF-LMS) with Fer-1 resulted in the downregulation of pro-ferroptotic genes such as TFRC, and the upregulation of anti-ferroptotic genes including GPX4, SLC7A11, FTL, and FSP-1 (Fig. 5B). These changes indicated the effective deactivation of ferroptosis-associated pathways in hCHF-LMS upon Fer-1 treatment. Additionally, LDH release assays performed with the supernatant of cultured hCHF-LMS showed decreased cell-injury in hCHF-LMS cultivated with Fer-1 (Fig. 5C). Furthermore, we evaluated the contractile function of hCHF-LMS in BMCC, and the results showed a significant increase in contractility in Fer-1 treated hCHF-LMS compared to the vehicle control group (Fig. 5D). On the contrary, the contraction and relaxation times remained unchanged (Supplementary fig. 4B). Unlike in the rat LMS (rAHF-LMS) experiments, Fer-1 treatment in hCHF-LMS did not have significant effects on the levels of interstitial collagen I or the expression of fibroblast activation genes (Supplementary fig. 4C–D).

Collectively, these findings indicate the significant involvement of ferroptosis-associated pathways in both acute and chronic heart failure. Treatment with Fer-1 improves cardiac contractile function and tissue viability in both settings. However, both rat models improvements in cardiac fibrosis, whereas the human chronic heart failure model, likely due to advanced heart failure stage, demonstrates elevated levels of fibroblast activation and collagen accumulation without significant response to Fer-1 treatment. This suggests that the effects of Fer-1 on cardiac tissue may vary depending on the specific pathological context, highlighting the regulation complexity of ferroptotic pathways in different stages of heart failure.

### 3.5 Targeting ferroptotic pathways deactivates cardiac fibroblasts and suppresses inflammatory response in vitro

**Fig. 6:**
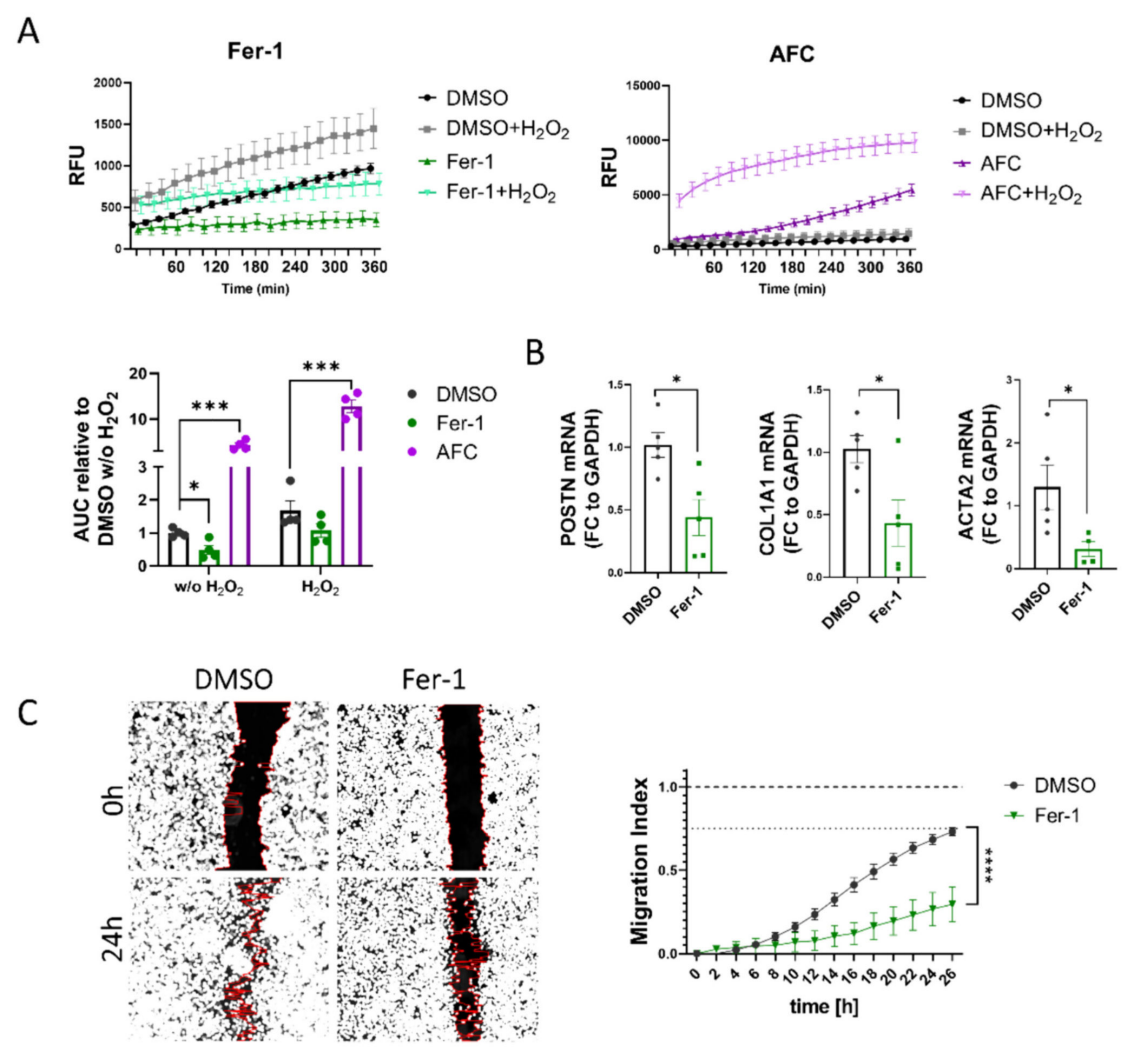
Inhibition of ferroptosis-related pathways *in vitro* exhibits anti-fibrotic and anti-inflammatory effects. (A) ROS production assay in human cardiac fibroblasts (HCF) over 6 h, with and without H_2_O_2_ stimulation. Fer-1 [10 μM] and AFC [100 μM] were used to inhibit or boost ferroptosis, respectively. Luminiscence was measured *via* Cytation 1 (BioTek) (*p < 0.05, ****p < 0.0001; two-way ANOVA; n = 4). (B) RT-qPCR quantification of gene expression (*p < 0.05; Student's t-test; *n* = 5). (C) Migration (scratch) assay to measure HCF migration index. Left - Representative images of 0 h and 24 h scratches under Fer-1 [10 μM] or DMSO (1:1000) administration. Right – Migration index over 26 h – measurement were done in 2 h intervals (****p < 0.0001; two-way ANOVA; n = 3). Data are displayed as mean ± SEM.

Considering the robust anti-fibrotic impact observed in rAHF-LMS with Fer-1 treatment and the existing evidence indicating the connection between ferroptosis-associated pathways and fibroblast activation in various pathological conditions, we conducted further investigations to explore its direct effects on cardiac fibroblasts in an *in vitro* setting. Human cardiac fibroblasts (HCFs) were treated with Fer-1 for 48 h and a subsequent reactive oxygen species (ROS) production assay revealed a notable reduction in the generation of ROS in the Fer-1-treated cells. This effect was less pronounced after the addition of hydrogen peroxide, which boosts ROS production (Fig. 6A). Consistent with this, dexrozaxone (DXZ), an alternative ferroptosis inhibitor that acts as an iron chelator, exhibited a reduction in ROS production, albeit to a lesser degree (Supplementary fig. 5 A). On the other hand, HCFs treated with ammonium ferric citrate (AFC), an activator of ferroptosis that induces intracellular iron overload, had extensively high ROS production both in the presence and absence of hydrogen peroxide (Fig. 6A).

Analysis of marker genes associated with fibroblast activation revealed a significant decrease in the expression of ACTA2, COL1A1, and POSTN transcripts following Fer-1 treatment (Fig. 6B). Moreover, to assess the impact of Fer-1 on fibroblast migratory function, a scratch assay was performed. The results revealed a lower migratory index in HCFs treated with Fer-1 compared to the vehicle control (Fig. 6C). Importantly, cell toxicity staining indicated that the ratio of dead to total cells remained unchanged with Fer-1 treatment, confirming that the observed reduction in migratory function of HCFs was not due to cell death (Supplementary fig. 6B). Similarly, HCF migratory index was found to be lower under DXZ administration (Supplementary fig. 6C).

Furthermore, an NF-κB luciferase assay was performed using reporter cells to assess NF-κB signalling under ferroptosis inhibition. This assay utilizes a luciferase reporter gene construct that contains the NF-κB response elements. Polyinosinic:polycytidylic acid (poly I:C) was used as an immunostimulant to boost NF-κB activation. NF-κB signalling was found to be significantly repressed with the administration of Fer-1 in a dose-dependent manner, particularly in the presence of poly I:C stimulation. Additionally, DXZ was employed and displayed a significant reduction in NF-κB activation under poly I:C stimulation, following a dose-dependent pattern, akin to Fer-1 (Supplementary fig. 5D). These findings highlight the potent anti-inflammatory properties associated with inhibition of ferroptotic pathways.

Taken together, these findings establish a strong link between ferroptosis-associated pathways and cardiac fibroblast activation, revealing a mechanistic association between these myocardial pathways. Specifically, Fer-1 treatment leads to reduced levels of fibrosis-related transcripts, mitigates oxidative stress, and hampers the migratory capacity of myofibroblasts. Moreover, the inhibition of ferroptotic pathways demonstrates anti-inflammatory effects, evident from decreased inflammation markers. These effects potentially contribute to the enhanced contractile function and increased force generation observed in both acute and chronic heart failure models in LMS.

### 3.6 Metallothioneins play a role in facilitating the anti-ferroptotic effects of Fer-1 in heart failure

**Fig. 7:**
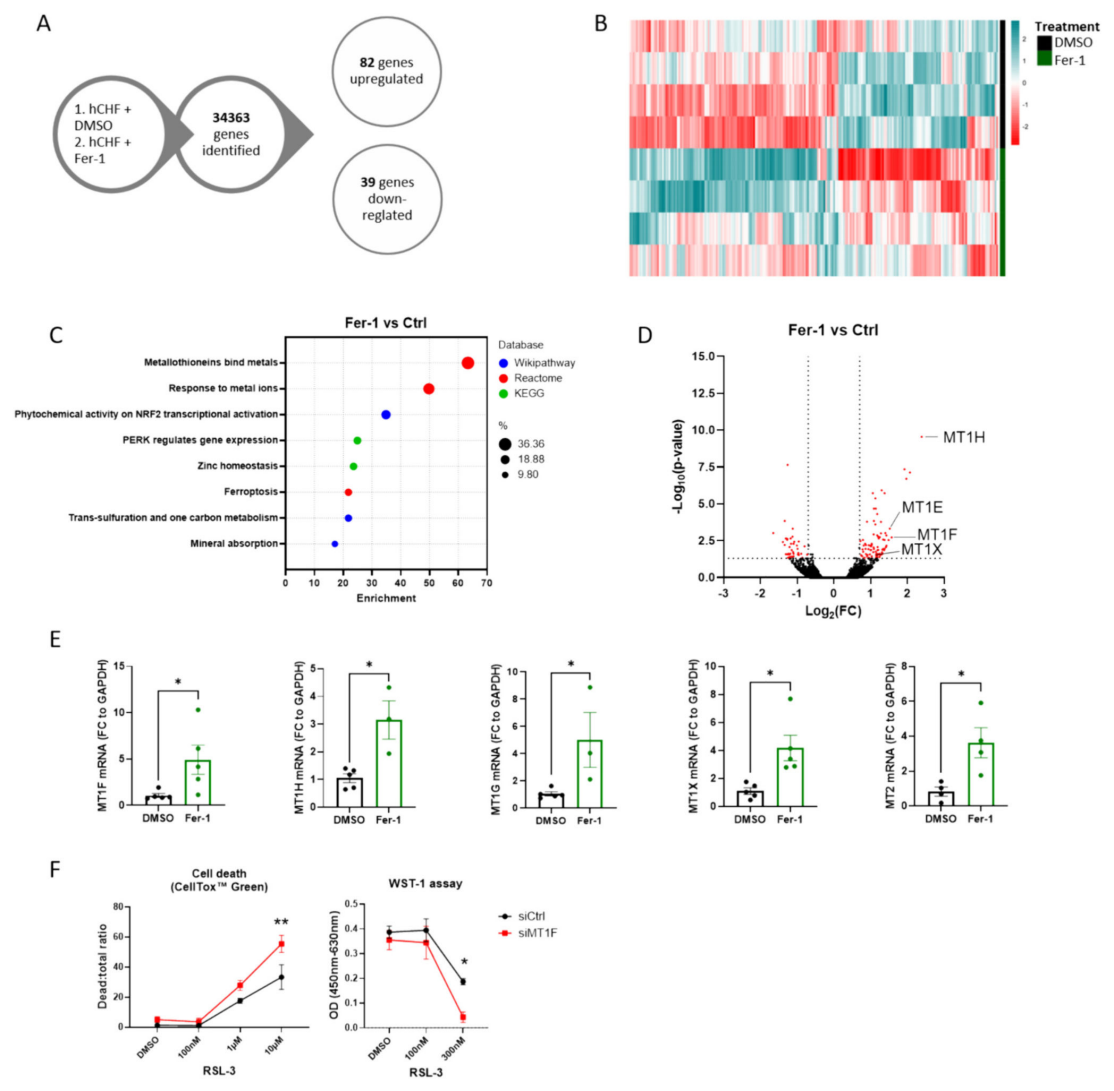
Metallothionein overexpression is associated with beneficial effects in human LMS and cardiac fibroblasts. (A) Diagram of RNA seq transcriptomic analysis. 2 groups were analyzed: hCHF-LMS treated with Fer-1 [10 μM] and hCHF-LMS trated with DMSO (1:1000) (p-ajd < 0.05; |Log_2_FC| > 0.5; n = 4). (B) Heatmap illustrating the expression of differentially expressed genes. Color scaling shows the levels for given proteins, blue - highest, red – lowest. (C) Functional enrichment analysis of RNA seq data. Selected clusters from different databases are included. X-axis - Normalized enrichment score hCHF+Fer-1 *vs*. hCHF+DMSO. FDR < 0.05. (D) Volcano plot of RNA seq dataset (p-ajd < 0.05; |Log_2_FC| > 0.7; n = 4). Differentially expressed Metallothionein 1 subtypes are highlighted. (E) RT-qPCR quantification of ferroptosis-related gene expression (*p < 0.05; Student's t-test; n = 5). Data are displayed as mean ± SEM. (F) Ferroptosis sensitization assays in HCF. Cell death was measured using CellTox™ Green or WST-1 following a 6-h treatment with increasing doses of RSL-3. (*p < 0.05; two-way ANOVA; n = 3). (For interpretation of the references to color in this figure legend, the reader is referred to the web version of this article.)

To investigate the molecular alterations occurring at the transcriptional level due to Fer-1 treatment, we conducted a high-throughput RNA-seq analysis of hCHF-LMS samples treated with either Fer-1 or vehicle control. The analysis included samples from four patients, revealing a total of 129 differentially expressed genes (DEGs), consisting of 85 upregulated genes and 44 downregulated genes (|log_2_FC| ≥ 0.5; padj≤0.05) (Fig. 7A-B, Supplementary table 3). We performed functional enrichment analysis using bioinformatics tools, focusing on pathway databases such as KEGG, Reactome, and Wikipathway (Fig. 7C). This analysis highlighted enrichment in the ferroptotic pathways, including key genes such as SLC7A11, SLC3A2, HMOX1, GCLM, and LPCAT3. Additionally, pathways related to ferroptosis initiation and propagation, such as “Phytochemical activity on NRF2 transcriptional activation” and “pERK regulates gene expression,” were also found to be enriched.

Of particular interest, the pathway showing the strongest enrichment was “Metallothioneins bind metals”, which encompasses genes such as MT1E, MT1F, MT1H, and MT1X from to the metallothionein family. Notably, MT1H displayed the highest upregulation in the dataset, with Log_2_FC = 2.39 (padj = 2.8 × 10^−10^). Enrichment was also observed in gene clusters associated with metallothionein function as heavy metal-binding proteins, including “response to metal ions”, “zinc homeostasis”, and “mineral absorption”. In order to investigate whether the upregulation of metallothioneins in hCHF-LMS could be attributed to cardiac fibroblasts, we conducted quantification of the different metallothionein subtypes in HCFs treated with Fer-1. Our analysis revealed a notable increase in the expression levels of MT1F, MT1H, MT1G, MT1X, and MT2 (Fig. 7E). The expression of MT1A, MT1E and MT3 remained unchanged (Supplementary fig. 6). To investigate the cardio-protective role of the of metallothioneins, HCFs were transiently transfected with MT1F siRNA (siMT1F; Santa Cruz) or Ctrl siRNA. HCFs were then stimulated with increasing concentrations of RSL-3 (Sigma) before cell viability and proliferation was assessed (Fig. 7F). Cell survival assays indicate an increased sensitization of HCFs transfected with siMT1F compared to the control group (siCtr) triggered by increasing RSL concentrations. This suggests that the overexpression of MT1F in cardiac fibroblasts exerts anti-ferroptotic effects, leading to fibroblast deactivation and ultimately providing a cardioprotective effect.

## 4 Discussion

Living myocardial slices are rapidly gaining popularity among researchers as an *ex vivo* tool for studying cardiac biology and pathophysiology. These 3D organomimetics retain the structural and functional characteristics of the intact heart, providing a more physiologically relevant context for studying cardiac function compared to isolated cells in 2D culture. This approach allows for a more comprehensive understanding of cardiac processes and can contribute to advancements in the field of cardiovascular research. Additionally, the ability to generate LMS from human specimens provides a clinically relevant multicellular human model for translational research and refines any experimental *in vivo* approach. In this study, we employed LMS to investigate both acute and chronic heart failure, aiming to develop new therapeutic approaches for these maladaptive conditions.

To explore the changes that occur during the acute/subacute phase of heart failure following cardiac injury, we applied the cryoinjury technique to induce damage to approximately 30 % of the tissue. Traditional methods to study myocardial injury, such as animal models of left anterior descending artery (LAD) ligation, have variable injury sizes due to anatomical differences, which results in varying phenotypes. In this regard, our model provides a simpler and more reproducible approach to replicate pathological stimuli *ex vivo*, without causing mortality, pain and suffer to animals around the time of the procedure. While the specific mechanism of tissue damage induction with cryoinjury differs from human heart disease, *in vivo* studies comparing cryoinjury-induced infarction to LAD-ligation in mice have demonstrated that the cryoinfarction model can be representative of infarcts observed in clinical practice. This model exhibits impaired cardiac function and histological changes, including CM hypertrophy and the deposition of collagenous fibers in and around the scar area [44,45]. Similarly to *in vivo* models of cardiac injury, our model demonstrated impaired contractile function, manifested by decreased contractility and prolonged time to 50 % peak, both of which suggest compromised systolic function. Additionally, we observed prolonged time to 90 % relaxation and increased tau, indicating impaired diastolic function. The observed changes are consistent with the findings of Dries et al., who noted electrophysiological alterations in rat LMS after cryoinjury. These alterations were characterized by impaired calcium transient, particularly in the subendocardial myocardium [15]. In addition, our model exhibited localized structural and morphometric remodelling of the surviving myocardium at the border zone of the cryoinjury. This included features such as CM hypertrophy and interstitial fibrosis, indicative of pathological changes in the tissue architecture. The observed hypertrophy of CMs was found to be limited to the remote regions and did not adequately compensate for the overall contractility produced by rAHF-LMS. This lack of compensation could be attributed to multiple factors, including the reduced number of contractile myocytes, the possible release of cytokines and inflammatory factors from injured cells, and the impaired calcium transient in the cells adjacent to injury, as previously reported [15]. Overall, these findings reflect the remodelling processes that occur in response to cardiac injury and support the relevance of our model in studying the associated alterations in the myocardium.

Unbiased proteomics analysis of injured rat LMS revealed the enrichment of pathways associated with various aspects of cardiac function and pathology, including dilated and hypertrophic cardiomyopathies, ECM organization, and energy production. These findings provide further validation for the resemblance of our model to cardiac injury observed *in vivo*. By comparing our proteomics data with a previously published RNA seq dataset from patients with DCM and HCM, we observed a significant overlap of deregulated genes and proteins. Although this comparison involves mRNA and protein datasets across different species, the consistent deregulation of these genes confirms the translatability and clinical relevance of our model. Moreover, it highlights a group of genes that likely play a significant role in the disease mechanisms and could serve as potential targets for therapeutic intervention.

Proteomics analysis provided evidence for ferroptosis-associated pathways, leading to an iron-dependent cell death mechanism related to lipid peroxidation, to be relevant in this model. Iron homeostasis in cardiac health and disease is extremely complex. Iron plays a crucial role in facilitating oxidation-reduction reactions that are vital for oxygen metabolism and oxidative phosphorylation in the heart [46,47]. On the other hand, the reactivity of free iron with oxygen leads to Fenton-type reactions, generating reactive oxygen species (ROS) that can damage lipids and lead to their peroxidation and, eventually ferroptosis and cell death [20,48]. Therefore, a fine balance of iron levels and distribution in the cell is necessary.

Interfering with ferroptosis is an emerging therapeutic approach for multiple pathological disorders. Here, we show that chemical inhibition of ferroptotic pathways by ferrostatin (Fer-1) in both the acute and chronic heart failure models, as well as, a non-injury based models using TGF-β1 stimulation results in cardioprotective effects. Fer-1 is a small molecule compound that acts as a powerful scavenger of lipid peroxides, effectively preventing the propagation of lipid oxidation reactions. By inhibiting lipid peroxidation, it helps to maintain the integrity of cell membranes and protect cells from ferroptotic cell death [49]. In both rat models, Fer-1 improved function, decreased fibroblast activation, and increased tissue viability, while in hCHF-LMS we saw increased contractility and viability. Fer-1 was found to deactivate human cardiac fibroblasts, indicating their potential role in mediating the overall beneficial effects observed in LMS. However, further research is needed to explore the specific impacts of ferroptosis blockers on CMs and other cell types within the heart. Interestingly, transcriptomic analysis of hCHF-LMS showed enrichment of the nuclear factor-erythroid 2-related factor 2 (NRF2) pathway, which is a master regulator of the antioxidant response and has been shown to regulate the activity of several ferroptosis and lipid peroxidation-related proteins in cancer and neurode-generative diseases [50,51]. This also aligns with the work conducted by Fang et al., which presented evidence supporting the role of ferroptosis in doxorubicin-induced cardiomyopathy through an NRF2-mediated mechanism, involving its downstream gene HMOX1 [52], which was also upregulated in our model, implying a potential convergence towards comparable pathways.

Metallothioneins (MT) are a family of cysteine-rich, low molecular weight proteins that have binding capacity to heavy metals, such as zinc and cadmium, through cysteine thiol groups [53]. Several subtypes of MT were upregulated in hCHF-LMS following Fer-1 treatment. Even though the role of MTs in ferroptosis is not fully understood, one of its subtypes – MT-1G – was found to negatively regulate ferroptosis in hepatocellular and renal cell carcinomas and to facilitate resistance to sorafenib treatment [54,55]. Reportedly, knockdown of MT-1G increases glutathione depletion and lipid peroxidation, therefore eventuating in ferroptosis and cell death [54]. We observed an increase in the expression of MT subtypes in human cardiac fibroblasts following Fer-1 treatment. Additionally, we found that inhibiting MT-1F expression with siRNA made human cardiac fibroblasts more sensitive to ferroptosis stimulation. These finding provide support for our hypothesis that fibroblasts play a major role in mediating the anti-ferroptotic effects of Fer-1. The upregulation of MT subtypes in fibroblasts suggests further their involvement in the cellular response to Fer-1 treatment, potentially contributing to the observed beneficial effects in mitigating ferroptosis.

Interestingly, MTs were recently found to be also regulated by Nrf2 activity [56]. Studies on diabetes- and intermittent hypoxia-induced cardiomyopathies have suggested a cardioprotective effect of MTs, associated with upregulation of Nrf2 and its downstream gene expression [53,56,57]. Our data thus suggest that Nrf2 is a master transcription factor involved in blocking ferroptosis in the heart by regulating genes involved in redox homeostasis and, among others, MTs. This is possibly achieved by the thiol activity of MTs, which contributes to maintaining glutathione (GSH) levels. GSH participates in the glutathione peroxidase (GPX) enzyme system, which helps to neutralize lipid peroxides and prevent their accumulation, thus inhibiting ferroptosis [58]. The activity of MT in neutralizing ROS contributes to the maintenance of cellular GSH levels. This, in turn, enhances the capacity of GSH to neutralize lipid peroxides and prevents the occurrence of ferroptosis. Moreover, thiols can directly scavenge lipid peroxides and ROS that contribute to ferroptosis initiation. By donating electrons, thiols can neutralize these reactive species and inhibit the propagation of lipid peroxidation [59].

## 5 Study limitations

While myocardial slices are organotypic, they exist as an isolated system, devoid of hormonal, neuronal, and inflammatory influences and their associated feedback loops [60], therefore, it is crucial to take this into consideration when interpreting the findings. Another factor to consider is the geometry of slices. When LMS contract, stress and strain vectors occur across a 2D plane, parallel to the direction of fibers. In contrast, the *in vivo* working heart experiences constant three-dimensional pressure and volume changes. To address this, the use of geometrical models that convert stress and strain to pressure and volume may help mitigate this difference. Furthermore, the investigation of physiological 3D model, such as the LMS, is also limited by its complexity. Well established assays, available for 2D cell culture, are not applicable for LMS or need adjustments of new protocols. Furthermore we want to clarify, that while we found ferroptotic pathways enriched in our comprehensive omics analyses in all used cardiac models and could mitigate cell death, fibrosis-associated gene expressions and cardiac contractility with a known ferroptosis inhibitor (Fer-1), our models do not necessarily show full ferroptotic phenotypes. Direct actions of Fer-1 on inhibiting lipid peroxidation and mitochondrial fitness could also explain the observed effects in our models. An additional limitation is that bulk RNA sequencing provides an average gene expression profile for the entirety of cells within LMS. Conversely, opting for single-cell sequencing allows for the examination of individual cells, revealing the heterogeneity within the cell population.

## 6 Conclusion

The current study presents new findings on the role of ferroptosis in both acute and chronic heart failure. Our research demonstrates that specifically targeting ferroptotic pathways in these conditions lead to significant cardioprotective effects, including improved contractile function, potentially through beneficial effects on fibrosis and inflammation. Additionally, our study herein provides the first transcriptomic analysis of inhibiting ferroptosis-associated pathways in the diseased human heart. Global transcriptome analysis reveals the involvement of Nrf2 and downstream MTs as potential mechanistic mediators and promising drug targets for future therapeutic approaches in human heart failure patients.

## Supplementary Material

Supplementary Material Supplementary data to this article can be found online at https://doi.org/10.1016/j.yjmcc.2024.09.012.

## Figures and Tables

**Table 1:** Antibodies and compounds used for immunofluorescence (IF) and western blot (WB).

Antibody/compound	Manufacturer	Catalog #	Origin	Dilution
Anti-Collagen I antibody	Abcam	ab34710	Rabbit	1:500 (IF)
Monoclonal Anti-alpha-Actinin (Sarcomeric)	Sigma Aldrich	A7811	Mouse	1:250 (IF)
Donkey anti-Mouse IgG, Alexa Fluor™ 488	Thermo Fisher	A21202	Donkey	1:2000 (IF)
Donkey anti-Mouse IgG, Alexa Fluor™ 546	Thermo Fisher	A10036	Donkey	1:2000 (IF)
Donkey anti-Rabbit IgG, Alexa Fluor™ 488	Thermo Fisher	A21206	Donkey	1:2000 (IF)
Donkey anti-Rabbit IgG, Alexa Fluor™ 546	Thermo Fisher	A10040	Donkey	1:2000 (IF)
Hoechst 33342	Invitrogen	H3570	–	1:1000 (IF)
Wheat Germ Agglutinin (WGA)	Invitrogen	W11261	–	1:1000 (IF)
Anti-MMP2 antibody	Abcam	ab37150	Rabbit	1:1000 (WB)
Anti-alpha smooth muscle Actin antibody	Abcam	ab5694	Rabbit	1:1000 (WB)
Anti-Galectin 3 antibody	Abcam	ab53082	Rabbit	1:1000 (WB)
Anti-Osteopontin antibody	Abcam	ab8448	Rabbit	1:1000 (WB)
Anti-rabbit IgG, HRP-linked Antibody	Cell Signalling	#7074	Goat	1:10000 (WB)

**Table 2:** RT-qPCR primer sequences. Rno - *rattus norvegicus*; hsa – *homo sapiens*.

Target	Forward primer sequence (5′ → 3′)	Reverse primer sequence (5′ → 3′)
rno_IL6	CTCTCCGCAAGAGACTTCCA	AGTCTCCTCTCCGGACTTGT
rno_MERTK	ATGCTCTTCTGGCCTCTGAG	CTCCCCTAGCCTCTGTGTTT
rno VIM	GGGAGGAGAGCAGGATTTCT	TCATCGTGGTGCTGAGAAGT
rno_POSTN	GTTCCTGTGTGACGTTGACC	CGGGGCAGCATTCATATAGC
rno_CTGF	GAAGCAGAGTCGTCTCTGCA	AGAAAGCTCAAACTTGACAGGC
rno_GAPDH	GAAGGGCTCATGACCACAGT	GGATGCAGGGATGATGTTCT
rno_FN1	GGATCCCCTCCCAGAGAAGT	GGGTGTGGAAGGGTAACCAG
rno_TGFß1	CCTGGAAAGGGCTCAACAC	CAGTTCTTCTCTGTGGAGCTGA
rno_COL1A1	ACGCATGGCCAAGAAGACAT	AAGCATACCTCGGGTTTCCA
rno ACTA2	CATCACCAACTGGGACGACA	TCCGTTAGCAAGGTCGGATG
hsa_COL1A1	ACGAAGACATCCCACCAATC	CTTGGTCGGTGGGTGACTCT
hsa_ACTA2	CCTGACTGAGCGTGGCTATT	GATGAAGGATGGCTGGAACA
hsa_MMP2	TGACATCAAGGGCATTCAGGAGC	GTCCGCCAAATGAACCGGTCCTTG
hsa_POSTN	TAGTCGTATCAGGGGTCGGG	TGGGCAGCCTTTCATTCCTT
hsa_GPX4	ATTGGTCGGCTGGACGAG	CCGAACTGGTTACACGGGAA
hsa_SLC7A11	TGGAACGAGGAGGTGGAGAA	TGGTGGACACAACAGGCTTT
hsa_FSP1	CTGTCTGTGAGGGCAGGAAG	CCCCTGCTAGCCGTGTAAAA
hsa_TFRC	GGACGCGCTAGTGTTCTTCT	GCTGTCCAGTTTCTCCGACA
hsa_FTL	GCCACTTCTTCCGCGAATTG	TCCAAAAGGGCCTGGTTCAG
hsa_MT1F	TGCAAGTGCAAAGAGTGCAA	CCCTTTGCAAACACAGCCC
hsa_MT1G	AAAGGGGCATCGGAGAAGTG	GCAAAGGGGTCAAGATTGTAGC
hsa_MT1H	CTGCAAAGGGGCGTCAGAGAA	GGAATGTAGCAAATGAGTCGGAGT
hsa_MT1X	CTGCTTCTCCTTGCCTCGAA	TGTCTGACGTCCCTTTGCAG
hsa_MT2	ATGCACCTCCTGCAAGAAAAG	GGTTTGTGGAAGTCGCGTT
hsa_GAPDH	CCAGGCGCCCAATACG	CCACATCGCTCAGACACCAT

## Abbreviations

AFC: Ammonium ferric citrate
AHF: Acute heart failure
BMCC: Biomimetic cultivation chambers
CHF: Chronic heart failure
CM: Cardiomyocyte
CSA: Cross-sectional area
CVD: Cardiovascular diseases
DCM: Dilated cardiomyopathy
DXZ: Dexrazoxane
ECM: Extracellular matrix
Fer-1: Ferrostatin 1
GPX4: Glutathione Peroxidase 4
GSH: Glutathione
HCF: Human cardiac fibroblasts
HCM: Hypertrophic cardiomyopathy
HF: Heart Failure
ICM: Ischemic cardiomyopathy
LMS: Living myocardial slice
LDH: Lactate Dehydrogenase
MT: Metallothionein
NRF2: Nuclear factor erythroid 2-related factor 2
Poly I:C: Polyinosinic: polycytidylic acid
ROS: Reactive oxygen species
TGF-β1: Transforming Growth Factor Beta 1
TUNEL: Terminal deoxynucleotidyl transferase dUTP Nick End Labeling
WST-1: Water-Soluble Tetrazolium
