# Supplementary material for: Ex vivo modelling of cardiac injury identifies ferroptosis-related pathways as a potential therapeutic avenue for translational medicine
Source: J Mol Cell Cardiol. Author manuscript; Available in PMC 2024 Dec 16. (PMC7617241; doi:10.1016/j.yjmcc.2024.09.012)
Supplement: Supplementary Material — Supplementary data to this article can be found online at https://doi.org/10.1016/j.yjmcc.2024.09.012. [file EMS200381-supplement-Supplementary_Material.zip › 1-s2.0-S0022282824001615-mmc2.docx]

***Ex vivo* modelling of cardiac injury identifies ferroptotis-related pathways as a potential therapeutic avenue for translational medicine**

Naisam Abbas (M.D., Ph.D.)^a,b,$^ and Marco Bentele ^a,$^, Florian J. G. Waleczek^a,b^, Maximilian Fuchs (M.Sc.)^b^, Annette Just^a^, Angelika Pfanne^a^, Andreas Pich (Ph.D.)^d^, Sophie Linke ^a^, Susanne Neumüller ^a^, Angelika Stucki‑Koch ^b^, Filippo Perbellini (Ph.D.)^a^, Christopher Werlein (M.D.)^e^, Wilhelm Korte (M.D.)^f^, Fabio Ius (M.D.)^f^, Arjang Ruhparwar (M.D.)^f^, Natalie Weber (M.D., Ph.D.)^a,^*, Jan Fiedler (Ph.D.)^b,^*, Thomas Thum (M.D., Ph.D.)^a,c,^*

^a^ Institute of Molecular and Translational Therapeutic Strategies (IMTTS), Hannover Medical School, Hannover, Germany
^b^ Fraunhofer Institute of Toxicology and Experimental Medicine (ITEM), Hannover, Germany
^c^ Center for Translational Regenerative Medicine, Hannover Medical School, Hannover, Germany
^d^ Institute of Toxicology and Core Unit Proteomics, Hannover Medical School, Hannover, Germany
^e^ Institute of Pathology, Hannover Medical School, Hannover, Germany
^f^ Department of Cardiothoracic, Transplantation and Vascular Surgery, Hannover Medical School, Hannover, Germany
^$^ These authors contributed equally and share the first authorship
*These authors contributed equally and share the last authorship

Supplementary information


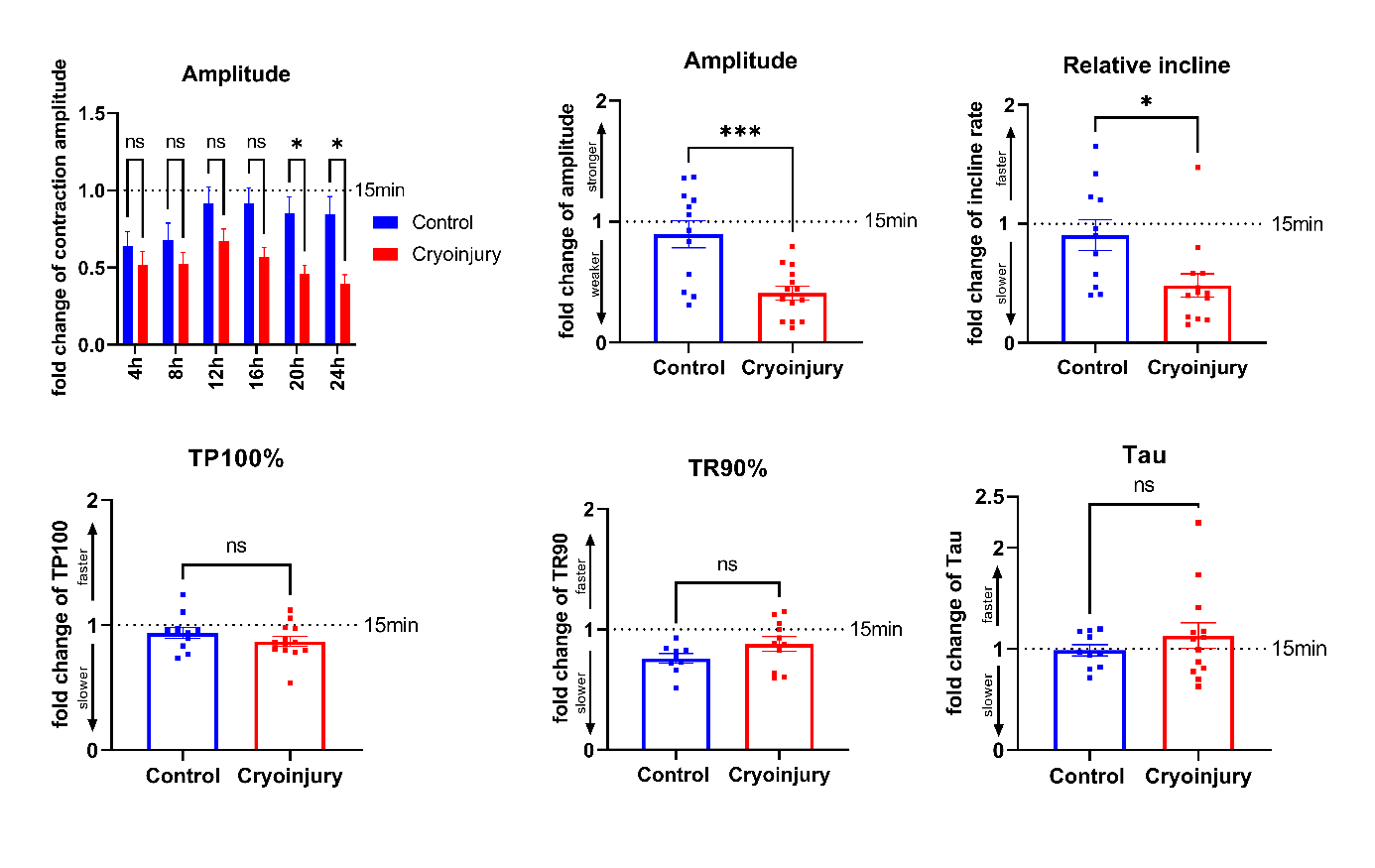


**Supplementary figure 1.** Contractile parameters of rCtrl-LMS and rAHF-LMS obtained from

BMCC.(*p<0.05; Student’s t test; n=6). TP – time to peak; TR – relaxation time. Data are displayed as

mean±SEM.


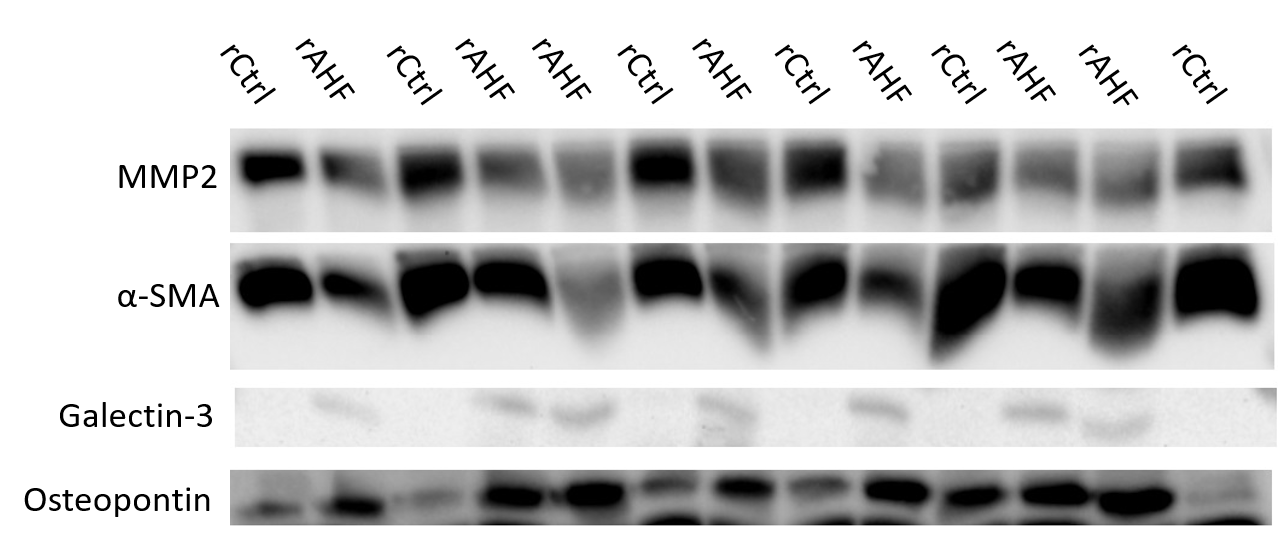


**Supplementary figure 2.** Western blot analysis of secreted proteins (MMP2, α-SMA, galectin-3 and

osteopontin) into the supernatant of rCtrl- and rAHF-LMS

**Supplementary figure 3.** Volcano plots of preoteomics dataset. Left – rCtrl vs. rAHF-Peri-injury. Right - rCtrl vs. rAHF-Remote (p-ajd<0.05; |Log₂FC|>0.7; n=3).


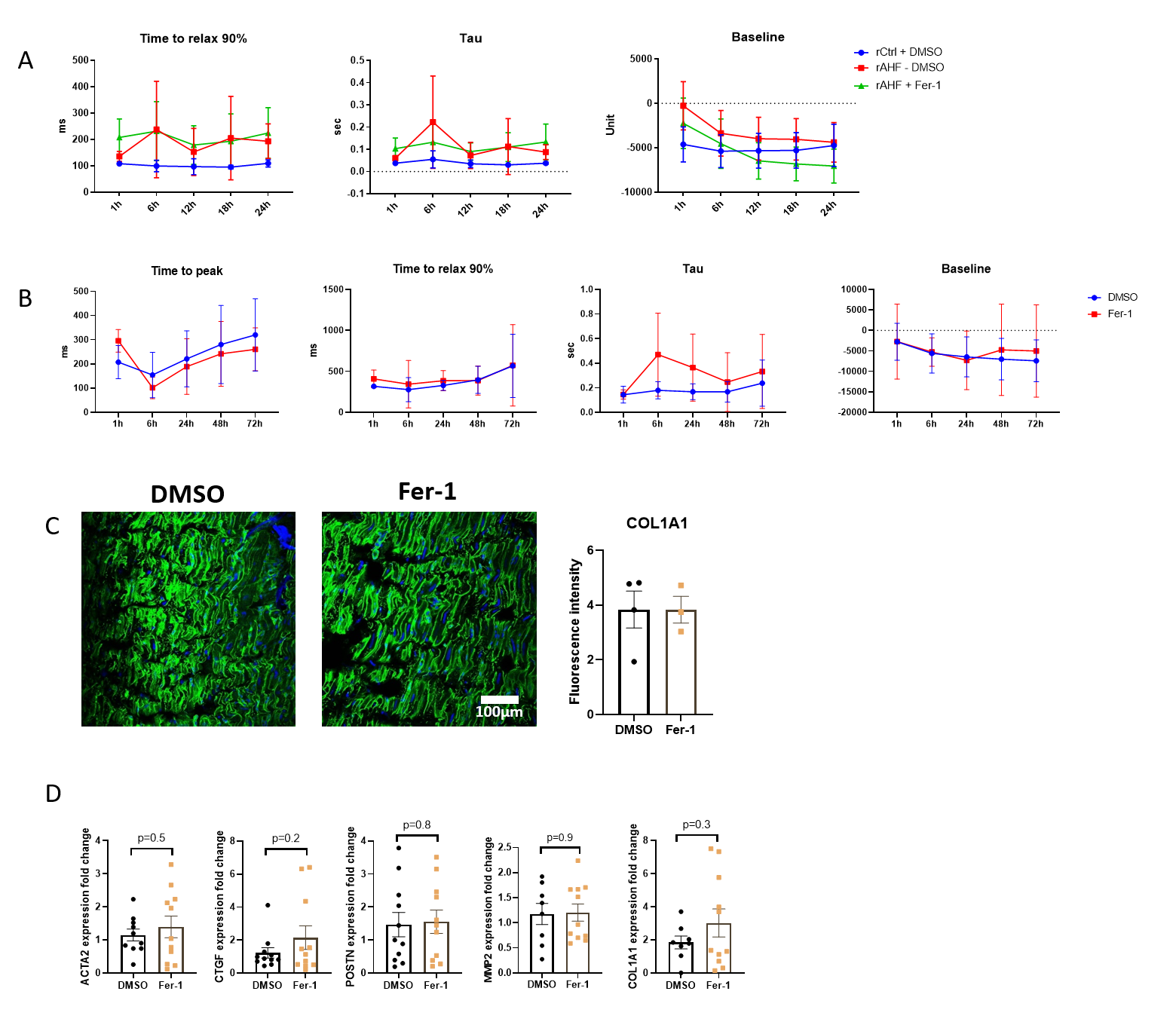


.

**Supplementary figure 4.** (A) Time course of relaxation parameters of rat LMS (rCtrl- and rAHF-LMS) ± Fer-1 [10µM] treatment during culture in BMCC: Time to 50% relaxation, Tau, baseline (two-way ANOVA; n=4). (B) Time course of contractile parameters of human LMS (hCHF-LMS) ± Fer-1 [10µM] treatment during culture in BMCC: Time to peak, Time to 90% relaxation, Tau, baseline (two-way ANOVA; n=5-6). (C) Representative images and quantification of collagen I immunostaining in hCHFLMS ± Fer-1 [10µM] treatment (Student’s t test, n=3-4). (D) RT-qPCR quantification of fibrosis-related gene expression in hCHF (Student’s t test; n=11). Data are displayed as mean±SEM.


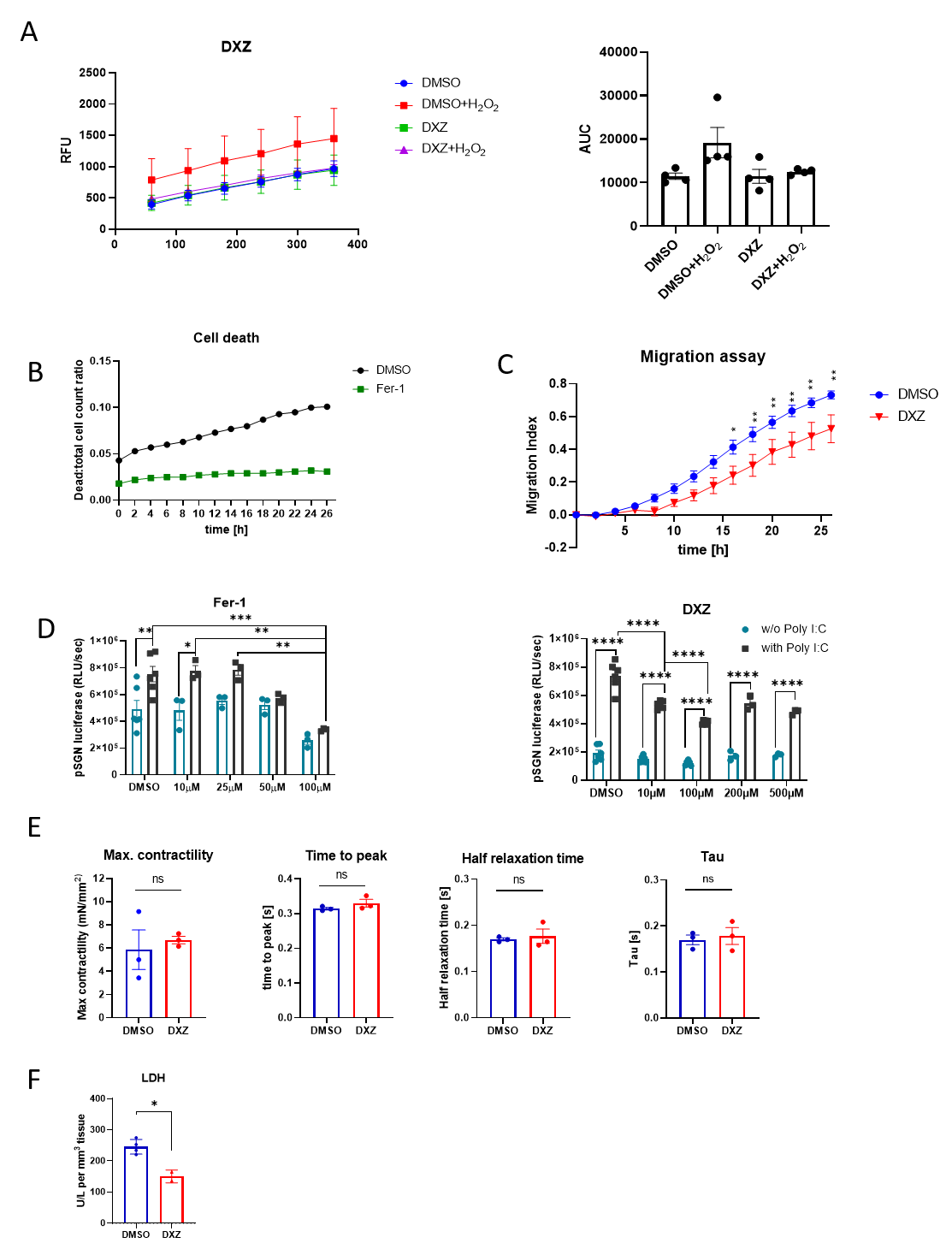


**Supplementary figure 5.** (A) ROS production assay in human cardiac fibroblasts over 6h, with and without H₂O₂ stimulation. DXZ [100µMwas used to inhibit ferroptosis. Right – comparison of AUC anlysis (one-way ANOVA; n=4). (B) Cell death quantification via CellTox cytotoxicity staining over 26h. (C) Migration index over 26h – measurement were done in 2h intervals (*p<0.05, **p<0.01; two-way ANOVA; n=3). (D) NF-κB reporter assay measuring the expression of NF-κB-dependent luciferase by in HEK293 cells, with and without poly I:C stimulation. (*p<0.05, **p<0.01, ***p<0.001, ****p<0.0001; two-way ANOVA; n=3). Data are displayed as mean±SEM. (E) Force measurement of LMS in the force transducer and quantification of contractile parameters: Maximal contractility, Time peak, Half relaxation time and Tau (τ; Student’s t test; n=3). Data are displayed as mean ± SEM. (F) LDH release measured from supernatants of human LMS cultured with or without DXZ ). (*p<0.05, ordinary one-way ANOVA; n=3).

**Supplementary figure 6.** RT-qPCR quantification of metallothionein gene expression in HCF cells (Student’s t test; n=5). Data are displayed as mean±SEM

**Supplementary table 1**: DAPs found in proteomics analysis: rAHF-P vs. rCtrl and rAHF-R vs. rCtrl. (|log2FC|≥0.5; pajd≤0.05; n=3)

| rAHF-Peri Injury vs. rCtrl | | rAHF-Remote vs. rCtrl | |
| --- | --- | --- | --- |
| Upregulated | Downregulated | Upregulated | Downregulated |
| RGD1565355 | Cox15 | Epb4.1 | Commd7 |
| Hist2h3c2;Hist1h3e | Sdhaf1 | Hmox2 | Cab39 |
| Epb4.1 | Ostf1 | Gmfb | Smim8 |
| Apoa1 | Acaa1a;Acaa1b | Mybbp1a | LOC100912478 |
| Ahsg | Bag5 | Ctsl | Blvra |
| Apoh | Clic5 | Parva | Smc1a |
| Hmox2 | Mrpl39 | Parl | Ehbp1l1 |
| H3f3b;H3f3c | Cbr1 | Hist2h3c2;Hist1h3e |  |
| Hist1h4b | Plaa | Plxnb2 |  |
| Ttr | Trim28 | Hnrnpa0 |  |
| F2 | Khdrbs1 | Rpl21 |  |
| Glg1 | Clip1 | Glg1 |  |
| Tmem182 | Gsk3b | RGD1565355 |  |
| H2afy | Psmd5 | Serhl2 |  |
| Fgg | Tollip | Nqo1 |  |
| Hpx | Smim8 | Plp2 |  |
| Hist1h2bk | Blvra | Hnrnpul2 |  |
| Fn1 | Ndufaf5 | Prkacb |  |
| Fgb | Bola3 | Adhfe1 |  |
| M0RAV0 | Cab39 | Dbnl |  |
| C3 | Ehbp1l1 | Gnb3 |  |
| Plp2 | Smc1a | Mtnd4l |  |
| Apoe |  | Bdh2 |  |
| Enpp1 |  | Eef1e1 |  |
| Serpina1 |  | Rhoc |  |
| Dnajc5 |  | Cnp |  |
| Fga |  | Crip1 |  |
| Ephx1 |  | Csrp2 |  |
| Plg |  | Tspan18 |  |
| Cd48 |  | Apoa1 |  |
| Mtdh |  | Ddx21 |  |
| Gc |  | Tagln |  |
| Tspan18 |  | Ddx19a |  |
| Mmgt1 |  | Hist1h1c |  |
| Tspan9 |  | Ccdc127 |  |
| Parva |  | Gna11 |  |
| Plxnb2 |  | Atl3 |  |
| Dhrs7b |  | Hist1h4b |  |
| Gnb1 |  | Dync1li2 |  |
| Sdcbp |  | Srsf1 |  |
| H2afz;H2afv |  | Rpl7a |  |
| Abhd12 |  | Arhgef1 |  |
| Icam2 |  | Agpat3 |  |
| Trim26 |  | Fundc1 |  |
| Tf |  | A0A0G2JW88 |  |
| Pcyox1l |  | Rer1 |  |
| Dhrs7 |  | Cd48 |  |
| Stx7 |  | Pcyox1l |  |
| Rer1 |  | Tmem261 |  |
| RT1-Bb |  | Icam2 |  |
| Ogn |  | Gnb1 |  |
| Art4 |  | Ogn |  |
| Mfge8 |  | Vti1b |  |
| Cp |  | Stx7 |  |
| Mtnd4l |  | Rcn1 |  |
| Apoa4 |  | S100a13 |  |
| M6pr |  | Rcsd1 |  |
| Napepld |  | Cd38 |  |
| Gsn |  | Pfn2 |  |
| Cd38 |  | Rnh1 |  |
| Cd34 |  | Lyn |  |
| Alb |  | M6pr |  |
| Fbn1 |  | Gpr107 |  |
| Chchd10 |  | Fhl2 |  |
| Gnb3 |  | Phospho1 |  |
| Clec10a |  | Gsta3 |  |
| Emcn |  | LOC100911683 |  |
| Cd151 |  | Bag5 |  |
| Ppap2a |  | Pds5b |  |
| Hk1 |  | Ap2m1 |  |
| Actn2 |  | Rpl7 |  |
| Bcl2l1;Bcl2l1-ps1 |  | Tmx3 |  |
| S100a13 |  | Abhd12 |  |
| Pcyox1 |  | Rpl4 |  |
| Serpina3k |  | Septin-9 |  |
| Itfg3 |  | Hist1h1e |  |
| Fundc1 |  | Mtdh |  |
| Tmed7 |  | Sco1 |  |
| Cnp |  | Snx12 |  |
| Lpl |  | Prpf19 |  |
| Cntnap3 |  | Rpl6 |  |
| Sirpa |  | Numa1 |  |
| Ppap2b |  | Psap |  |
| Mybbp1a |  | Bche |  |
| Gpd2 |  | Usp9x |  |
| Enpp4 |  | Gnaq |  |
| Lrp1 |  | Atp5e;LOC100361879 |  |
| Selenoi |  | Tmem182 |  |
| Rpl7a |  | Hist3h2bb |  |
| Dbnl |  | Nolc1 |  |
| Tmx3 |  | Csrp1 |  |
| Myh10 |  | Ppp2r5c;LOC100909464 |  |
| Xpnpep2 |  | Coq10b |  |
| Cpne1 |  | Fgb |  |
| Tmed4 |  | H2afz;H2afv |  |
| Arhgef1 |  | Myoz2 |  |
| Agrn |  | Stxbp1 |  |
| Hist1h1e |  | Chmp6 |  |
| Ctsl |  | Dhrs7 |  |
| Cd59 |  | Cast |  |
| Entpd2 |  | Cdh5 |  |
| Esyt1 |  | Hbe1 |  |
| Cd36;LOC685953 |  | Dhrs7b |  |
| Itga7 |  | AI314180 |  |
| Endod1 |  | Cd59 |  |
| Chmp6 |  | Chpt1 |  |
| Serhl2 |  | Cbx3 |  |
| Hist1h1c |  | Mxra7 |  |
| Mtfp1 |  | Enpp1 |  |
| Lmod2 |  | H3f3b;H3f3c |  |
| Sptan1 |  | Rbbp4 |  |
| Tagln |  | S100a4 |  |
| Ptgfrn |  | Denr |  |
| Ccdc167 |  | Dnajc5 |  |
| Cyb5a |  | Sqstm1 |  |
| Alpl |  | Flna |  |
| Cebpzos |  | Mmgt1 |  |
| Gpr116 |  | Fxyd1 |  |
| Srsf1 |  | Dctn5 |  |
| A1m |  | Inpp1 |  |
| Prkacb |  | Myo1b |  |
| Rps6 |  | Kpna4 |  |
| Ca4 |  | Abcb10 |  |
| Serpinc1 |  | Ahsg |  |
| Igf2r |  | Hpx |  |
| Tfrc |  | Smc3 |  |
| Cd47 |  | Lrp1 |  |
| Itga1 |  | Rpl18 |  |
| Slc44a2 |  | Ppap2a |  |
| Aldh3a2 |  | Thy1 |  |
| Eef1e1 |  | Itfg3 |  |
| Rpl18 |  | H6pd |  |
| Nedd4l |  | Rpl13a |  |
| Ccdc127 |  | Gc |  |
| Tmem261 |  | Rhoa |  |
| Nqo1 |  | Cd36;LOC685953 |  |
| Dag1 |  | Sirt3 |  |
| Cst3 |  | Mfge8 |  |
| Lamp1 |  | Sncg |  |
| Fam210a |  | Psmb9 |  |
| Gpx3 |  | Src;Fyn;Yes1;Lck;Hck |  |
| Bcap29 |  | Ktn1 |  |
| Pigs |  | Vamp3 |  |
| Cd81 |  | Epb41l2 |  |
| Ermp1 |  | Fam120a |  |
| Anpep |  | Cox6a1 |  |
| Cr1l |  | Skp1 |  |
| Thy1 |  | Gsn |  |
| Ccdc176 |  | Tardbp |  |
| Eng |  | Tgm2 |  |
| Derl1 |  | Gng2 |  |
| Itgav |  | Prkcdbp |  |
| Entpd1 |  | Pgm2 |  |
| Sts |  | Rps6 |  |
| Nt5e |  | Cnn3 |  |
| Surf4;Surf1 |  | Agk |  |
| Clec2d11 |  | Esyt1 |  |
| Bgn |  | Chchd7 |  |
| Cd9 |  | Mt-atp6 |  |
| Sypl1 |  | Mcam |  |
| Dpp4 |  | Sub1 |  |
| Col6a1 |  | Hist1h1b |  |
| Ctsz |  | Rab5a |  |
| Bche |  | Mtnd3 |  |
| Rpl6 |  | Mrpl39 |  |
| Mrpl27 |  | Smarcc2;Smarcc1 |  |
| Rtn4 |  | Parp1 |  |
| Gpr107 |  | Capza1 |  |
| Enpep |  | Ctsz |  |
| Dcn |  | LOC683884 |  |
| Tspan8 |  | Rab5b |  |
| Atl3 |  | Txn2 |  |
| Myh14 |  | Hmgn2;LOC100360316 |  |
| Stom |  | Tpm3 |  |
| Chchd7 |  | Syngr2 |  |
| Tmem109 |  | Cmc2 |  |
| Ndufa11 |  | Rrbp1 |  |
| Col6a2 |  | Col4a2 |  |
| Des |  | Ctsb |  |
| Mp68 |  | Asph |  |
| Tmed5 |  | Fxn |  |
|  |  | Zfp692 |  |
| Pxmp2 |  | Cox6c2 |  |
| Lamc1 |  | Nenf |  |
| Ddx19a |  | Cox5a |  |
| Scarb2 |  | Mpc1 |  |
| Abcb10 |  | Sypl1 |  |
| Lman1 |  | Mp68 |  |
| Susd2 |  | Rps11 |  |
| Mtco3 |  | Actb |  |
| Lama2 |  | Ephx1 |  |
| Rhoc |  | Rpl15 |  |
| Usmg5 |  | Minos1 |  |
| Podxl |  | Tmed4 |  |
| Adhfe1 |  | Hnrnpa2b1 |  |
| Fis1 |  | Cox6b1 |  |
| Anxa3 |  | Sirpa |  |
| Hspg2 |  | Vdac3 |  |
| Plgrkt |  | Cox6a2 |  |
| Aqp1 |  | Add3 |  |
| Cspg4 |  | Arf4 |  |
| Apmap |  | Ndufv1 |  |
| Cyb5b |  | Bcl2l1;Bcl2l1-ps1 |  |
| Hnrnpa0 |  | Cmpk2 |  |
| Pecam1 |  | Ckmt2 |  |
| Csnk2b |  | Ppp1r12b |  |
| Gng2 |  | Ndufb2 |  |
| Lama4 |  | Ehd2 |  |
| Cox20 |  | Cspg4 |  |
| Syngr1 |  | Calr |  |
| Gmfb |  | Actg1 |  |
| Fignl1 |  | Ralb |  |
| Gpc4 |  | Septin-11 |  |
| Tmed2 |  | Cd47 |  |
| Tmem126a |  | Cebpzos |  |
| Agk |  | Reep5 |  |
| Synj2bp |  | Sdcbp |  |
| Anxa5 |  | Ptma |  |
| Reep5 |  | Clec10a |  |
| Rcn1 |  | Nedd4l |  |
| Vdac3 |  | Mtfp1 |  |
| Sco1 |  | Sars |  |
| Cav3 |  | Rps26 |  |
| Pln |  | Cd99 |  |
| Rps11 |  | Atp1b3 |  |
| Fbln5 |  | Acbd3 |  |
| Myl2 |  | Tagln2 |  |
| Itga5 |  | Surf4;Surf1 |  |
| Lamb1 |  | Lmf2 |  |
| Cdh13 |  | Cox17 |  |
| Cox6c2 |  | Rpl30 |  |
| Dctn5 |  | Ada |  |
| Vamp3 |  | Fkbp1a |  |
| Cd200 |  | Mrpl27 |  |
| Anxa4 |  | Myl2 |  |
| Chpt1 |  | Ehd1 |  |
| Anxa2 |  | Tjp1 |  |
| Selt |  | Ccdc176 |  |
| Minos1 |  | Fgg |  |
| Ndufb2 |  | LOC100912599 |  |
| Col4a2 |  | Gpd2 |  |
| Tmod1 |  | Cox20 |  |
| Slc25a11 |  | Cap1 |  |
| Bsg |  | Itga7 |  |
| Rtn3 |  | Pi4ka |  |
| Cd99 |  | Tf |  |
| Psmb9 |  | Tpm4 |  |
| Anxa1 |  | G3bp2 |  |
| Rlc-a |  | Lamp1 |  |
| Etfdh |  | Septin-8 |  |
| Pi4ka |  | Khdrbs1 |  |
| Fam162a |  | Sts |  |
| Vti1b |  | Fn1 |  |
| Atp5e;LOC100361879 |  | Cp |  |
| Capza1 |  | Clic1 |  |
| Capzb |  | LOC684828 |  |
| Bcap31 |  | Ptrf |  |
| Banf1 |  | Tmpo |  |
| Myl6 |  | Rtn4 |  |
| Itga6 |  | Rexo2 |  |
| Rpl21 |  | Stx12 |  |
| Hist1h1b |  | Bcl2l13 |  |
| Itgb1 |  | Hmx1 |  |
| Vdac1 |  | Podxl |  |
| RGD1565410;LOC100911104 |  | LOC685596 |  |
| Phb2 |  | Vim |  |
| Car14 |  | Atp5d |  |
| Cacna2d1 |  | Ccbl1 |  |
| Vnn1 |  | Mtco3 |  |
| Mtnd3 |  | Mt-Cyb |  |
| Cox5a |  | Tfam |  |
| Mtch1 |  | Lamtor3 |  |
| Lum |  | Alb |  |
| S100a10 |  | Ndufa6 |  |
| Enpp3 |  | Gstp1 |  |
| Rplp1 |  | Ndufa5 |  |
| Crip1 |  | Coq5 |  |
| Anxa7 |  | Timm8a;Timm8a1 |  |
| Rpl10;Rpl10l |  | Pgrmc1 |  |
| Lnpep |  | Pccb |  |
| Lamb2 |  | Pars2 |  |
| Erlin2 |  | Ilk |  |
| Lmf2 |  | Rpl10;Rpl10l |  |
| Ghitm |  | Ndufv3 |  |
| Parl |  | Akr1c9 |  |
| Cox6a2 |  | Ywhaq |  |
| Mt-atp6 |  | Aldh3a2 |  |
| Coq7 |  | Hmg1l1;Hmgb1;Hmgb1-ps3 |  |
| Myoz2 |  | Tmem33 |  |
| Col15a1 |  | Taldo1 |  |
| Lamp2 |  | Myl6 |  |
| Canx |  | LOC681355 |  |
| Pdcd6 |  | Eef1a1 |  |
| Flot2 |  | Cdnf |  |
| Rcsd1 |  | Strn3;Strn4 |  |
| RGD1565784 |  | Atp5l |  |
| Fxyd1 |  | Sptb |  |
| Rtn4 |  | Bgn |  |
| Asph |  | Q642A4 |  |
| Rpl30 |  | Fahd1 |  |
| Fhl2 |  | Lmnb1 |  |
| Ndufb7 |  | C1qbp |  |
| Rpl13a |  | Ppia |  |
| Actn4 |  | Ssbp1 |  |
| Gpc1 |  | Sri |  |
| Zfp692 |  | Ndufa8 |  |
| Cox6a1 |  | Esam |  |
| Phb |  | Ptbp1 |  |
| Sri |  | Serpina1 |  |
| Sirt3 |  | Eif3f |  |
| Flot1 |  | Psmb10 |  |
| Rab6a |  | Acaa1a;Acaa1b |  |
| Rpl15 |  | Pcyox1 |  |
| Mt-Cyb |  | Ssr4 |  |
| Csrp2 |  | Rps16 |  |
| Cox7a2 |  | Actr1a |  |
| Gnaq |  | Slc9a3r2 |  |
| Psap |  | Myh10 |  |
| Bcam |  | Cd151 |  |
| Pgrmc1 |  | Gimap4 |  |
| Cdh2 |  | Tmed5 |  |
| Nrp1 |  | Q68FZ8 |  |
| Ndufb9 |  | Usmg5 |  |
| Tomm40 |  | Entpd2 |  |
| Slmap |  | Pecam1 |  |
| Gng12 |  | Rps2;Gm8225 |  |
| Sptbn1 |  | Lta4h |  |
| Tmem43 |  | Map4 |  |
| Acta1 |  | Tpt1 |  |
| Coa3 |  | Actr3 |  |
| LOC684828 |  | Fam210a |  |
| Hnrnpul2 |  | Rtn3 |  |
| Sqrdl |  | Ak2 |  |
| Mxra7 |  | S100a10 |  |
| Atp5i |  | Cav1 |  |
| Cpne3 |  | Fbln5 |  |
| LOC100911130 |  | Clip1 |  |
| Ociad1 |  | Derl1 |  |
| Esam |  | Q9R1T1 |  |
| Cav2 |  | A0A0G2JUD4 |  |
| Flna |  | LOC100361144;LOC100362391 |  |
| Jagn1 |  | Rps18 |  |
| Anxa6 |  | Car14 |  |
| Sdhc |  | Cnpy2 |  |
| Atp5l |  | Pcca |  |
| Tmem33 |  | Hspg2 |  |
| Ncam1 |  | Anxa7 |  |
| Prelp |  | Gbas |  |
| Capza2 |  | Mgll |  |
| Mtco2 |  | Capns1 |  |
| Dync1li2 |  | Tspan9 |  |
| Ncstn |  | Fth1 |  |
| RGD1302996 |  | Cyb5a |  |
| Myl3 |  | Sspn |  |
| Coq10b |  | Rpl19 |  |
| LOC100361144;LOC100362391 |  | Lmod2 |  |
| Bdh2 |  | Fam162a |  |
| Calr |  | Snx2 |  |
| LOC100363239 |  | Rap1b |  |
| Ndufb5 |  | Lman2 |  |
| Rtn1 |  | Rps24 |  |
| Ndufb10 |  | Cat |  |
| LOC683884 |  | Lman1 |  |
| Apoo |  | Stoml2 |  |
| Lman2 |  | Cox5b |  |
| Ndufb11 |  | Lactb2 |  |
| Pfn2 |  | Hnrnpd |  |
| Sub1 |  | LOC100912534 |  |
| Rps26 |  | Cyc1 |  |
| Adipoq |  | Ca2 |  |
| Smim12 |  | Tln1 |  |
| Bcs1l |  | Rasip1 |  |
| Rps15a;Rps15al2 |  | Ndufa9 |  |
| Atp1b3 |  | Dpp7 |  |
| Sspn |  | Cbr1 |  |
| Lamtor3 |  | Ca4 |  |
| Rap1a |  | Ndufb10 |  |
| Nid1 |  | Col6a1 |  |
| Gbas |  | Add1 |  |
| Csnk2a1 |  | Pnp |  |
| Pgrmc2 |  | Tfrc |  |
| Rpl19 |  | Ccdc167 |  |
| Ralb |  | mrpl11 |  |
| Rpl4 |  | Ndufb7 |  |
| Napa |  | Rpl34;Rpl34-ps1 |  |
| Vcan |  | Ndufs4 |  |
| Cisd2 |  | Got2 |  |
| Rhoa |  | Lamc1 |  |
| Rap1b |  | Timm21 |  |
| Ndufb6 |  | Capzb |  |
| Rab5a |  | Mtch1 |  |
| Asah1 |  | Me3 |  |
| Tmem47 |  | Rps14 |  |
| Cox4i1 |  | Anxa11 |  |
| Csrp1 |  | Ssb;LOC680385 |  |
| Art3 |  | Ctsd |  |
| Nceh1 |  | Tmem126a |  |
| Cisd1 |  | Anp32b |  |
| Apool |  | Nudt21 |  |
| Ndufb1 |  | Col6a2 |  |
| Cdipt |  | Actn4 |  |
| S100a4 |  | Magohb;Magoh |  |
| Vps13a |  | Nfu1 |  |
| Tmem70 |  | Anxa5 |  |
| Rps24;LOC100363469 |  | Fga |  |
| Ndufa8 |  | Vdac1 |  |
| Rpl38;RGD1561636 |  | Tmx4 |  |
| Ssr4 |  | Cav3 |  |
| Fundc2 |  | Idh3a |  |
| Syngr2 |  | Rpl27;RGD1563835 |  |
| Stt3a |  | Ppp3ca;Ppp3cb |  |
| Vdac2 |  | Sdpr |  |
| Usp9x |  | Hadh |  |
| Pon2 |  | Psmd7 |  |
| mrpl11 |  | LOC684270 |  |
| Ndufa4 |  | Slc44a2 |  |
| Rps25 |  | Elavl1 |  |
| Septin-10 |  | Sdhaf1 |  |
| Gfer |  | Txn;Txn1 |  |
| Rnh1 |  | Marcks |  |
| Psmb10 |  | Bphl |  |
| Timm22 |  | Ppif |  |
| Ddx21 |  | Tmed7 |  |
| Rps18 |  | Cr1l |  |
| Vat1 |  | Apmap |  |
| Anxa11 |  | Aldh1a1 |  |
| Timm8a;Timm8a1 |  | Tmed2 |  |
| Vdac3 |  | Msn |  |
| Dhodh |  | Nit2 |  |
| Cav1 |  | Clic5 |  |
| Tmed10 |  | Atp5f1 |  |
| Timm9 |  | Vat1 |  |
| Pam16 |  | Lamb1 |  |
| Mcam |  | Ddost |  |
| Pyroxd2 |  | Akr1a1 |  |
| Snrpd1 |  | Ppp1ca |  |
| Higd1a |  | Phb2 |  |
| LOC685596 |  | Cd200 |  |
| Tmem256 |  | Apoh |  |
| Mgst3 |  | Napa |  |
| Ndufaf3 |  | Sgca |  |
| Tmem65 |  | Dpysl2 |  |
| Ctsb |  | Cpne3 |  |
| Dpp7 |  | Tpm1 |  |
| Rpl7 |  | Sdhc |  |
| Gypc |  | Anxa2 |  |
| Lyn |  | Uqcrh |  |
| Atp5j2 |  | Mtch2 |  |
| Mtco1 |  | Gstm1 |  |
| LOC100911483;Ndufa13 |  | Glb1 |  |
| Stoml2 |  | Fscn1 |  |
| Hpcal1;Hpca |  | Smim12 |  |
| Uqcr10 |  | Capza2 |  |
| Actg1 |  | Trim28 |  |
| Cisd3 |  | Ostf1 |  |
| Gna11 |  | Anxa3 |  |
| Timm23 |  | Prdx1 |  |
| Atp5f1 |  | Itga1 |  |
| Cox6b1 |  | Cfl1 |  |
| Slc25a21 |  | Clybl |  |
| Tmem14c |  | Rps12 |  |
| Gsta3 |  | Eef1b2 |  |
| Map4 |  | Vps13a |  |
| Cyc1 |  | Uqcrc1 |  |
| Mt-atp8 |  | Actr1b |  |
| Eif3f |  | Hrc |  |
| Cpt1a |  | Dld |  |
| Ptges2 |  | Tpm1 |  |
| Mtnd4 |  | Ppic |  |
| Rbbp4 |  | Emc2 |  |
| B2m |  | Arpc2 |  |
| Agpat3 |  | Bsg |  |
| LOC688684;Rpl32 |  | Agrn |  |
| Cap1 |  | Lgals1 |  |
| Ndufa5 |  | Bcap31 |  |
| Cdh5 |  | Etfdh |  |
| Ndufa6 |  | Tomm40 |  |
| Timm21 |  | Asah1 |  |
|  |  | Hibch |  |
|  |  | Mdh2 |  |
|  |  | Tomm22 |  |
|  |  | Lamp2 |  |
|  |  | Gk |  |
|  |  | Rab11a |  |
|  |  | Snrpd1 |  |
|  |  | Adck3 |  |
|  |  | Lama2 |  |
|  |  | Plbd1 |  |
|  |  | Arpc3 |  |
|  |  | Ndufb9 |  |
|  |  | Rpl11 |  |
|  |  | Stt3a |  |
|  |  | Pln |  |
|  |  | Rap1a |  |
|  |  | Casq2 |  |
|  |  | Slc25a11 |  |
|  |  | Myh14 |  |
|  |  | Ndufs7 |  |
|  |  | Coq9 |  |
|  |  | Gnai2 |  |
|  |  | Akr1cl |  |
|  |  | Pcmt1 |  |
|  |  | Itgav |  |
|  |  | Dag1 |  |
|  |  | Art3 |  |
|  |  | Gfer |  |
|  |  | Uqcrc2 |  |
|  |  | Anp32a |  |
|  |  | Atp1b1 |  |
|  |  | Bcap29 |  |
|  |  | Ndufa11 |  |
|  |  | Vdac2 |  |
|  |  | Cycs;LOC679794 |  |
|  |  | Sgcg |  |
|  |  | Cd9 |  |
|  |  | Hsd17b10 |  |
|  |  | Nutf2 |  |
|  |  | Ywhae |  |
|  |  | Csnk2a1 |  |
|  |  | Dysf |  |
|  |  | Pgd |  |
|  |  | Acta1 |  |
|  |  | Glod4 |  |
|  |  | Tspan8 |  |

**Supplementary table 2:** Supplementary table 2: Clinical information of the patients enrolled in the human heart failure LMS experiments.

| Patient | Age | Sex | Diagnosis | EF% |
| --- | --- | --- | --- | --- |
| 1 | 11 | male | DCM | LVEDD 60 |
| 2 | 61 | male | ICM | 10 |
| 3 | 60 | female | ICM | 50 |
| 4 | 20 | female | CHF following myocarditis | <10 |

DCM – dilated cardiomyopathy; ICM – ischemic cardiomyopathy, CHF – congestive heart failure

**Supplementary table 3:** DEGs found in RNA seq analysis: hCHF +Fer-1 vs. hCHF DMSO (|log2FC|≥0.5; pajd≤0.05; n=4).

| hCHF: Fer-1 vs. DMSO | |
| --- | --- |
| Upregulated | Downregulated |
| MT1H | DDO |
| GPRC5A | LMCD1 |
| RHCG | PDCD2L |
| HAS1 | NPR3 |
| HID1 | DSG2 |
| SLC3A2 | AC022034.1 |
| IL1RL1 | SSX2IP |
| SLC7A5 | VSIR |
| KLF4 | LINC01936 |
| BAIAP2 | PLCXD3 |
| SESN2 | MTND2P28 |
| ATF3 | CAVIN4 |
| DERL3 | PKP1 |
| DDIT3 | RANBP6 |
| MT1E | MT-ATP8 |
| HIF1A-AS3 | MT-ND2 |
| GABRR2 | MT-ND3 |
| STX3 | MT-ND1 |
| CTH | E2F8 |
| LSMEM1 | H19 |
| PTPRN | AC103740.1 |
| GEM | MT-TL1 |
| MT1F | MT-ND4L |
| ZNF469 | RAPGEF4 |
| ULBP1 | IGF2 |
| RND1 | AL365434.1 |
| LINC00520 | MT-ATP6 |
| CBX4 | RGS5 |
| SMG1P7 | KLHL38 |
| SOX30 | MTATP6P1 |
| CDC6 | HIGD1B |
| UAP1L1 | CA4 |
| SCX | MT-CO1 |
| LMNTD2-AS1 | MT-CO3 |
| SMOX | MT-ND4 |
| LURAP1L-AS1 | MT-ND5 |
| PDIA2 | CX3CL1 |
| NR4A2 | CD300LG |
| MAFA | GPIHBP1 |
| ZNF425 | ALDH1B1 |
| HMOX1 | FKBP5 |
| LRP2BP | PALLD |
| LINC00862 | SORBS1 |
| MANF | LPCAT3 |
| ESM1 |  |
| KCNQ1OT1 |  |
| SLC30A2 |  |
| TMEM63C |  |
| CHAC1 |  |
| HERPUD1 |  |
| AC068580.1 |  |
| ASPRV1 |  |
| BHLHA15 |  |
| TRIM36 |  |
| LRRC15 |  |
| FAM222A |  |
| MT1X |  |
| ENPP1 |  |
| MMP10 |  |
| FOSB |  |
| C2CD4A |  |
| AC144831.1 |  |
| VASN |  |
| RETREG1 |  |
| NR1D2 |  |
| FAM107B |  |
| ZSCAN5A |  |
| MXD1 |  |
| BEST1 |  |
| SRPK3 |  |
| HSPA1A |  |
| PMEPA1 |  |
| CEBPG |  |
| MTHFD2 |  |
| ANKRD37 |  |
| SLC7A11 |  |
| KRT8P12 |  |
| TP53INP2 |  |
| ZBTB21 |  |
| NFKBIZ |  |
| FAM117A |  |
| MAFB |  |
| EIF2AK3 |  |
| GCLM |  |
| SHMT2 |  |
