# Supplementary material for: Ex vivo modelling of cardiac injury identifies ferroptosis-related pathways as a potential therapeutic avenue for translational medicine
Source: J Mol Cell Cardiol. Author manuscript; Available in PMC 2024 Dec 16. (PMC7617241; doi:10.1016/j.yjmcc.2024.09.012)
Supplement: Supplementary Material — Supplementary data to this article can be found online at https://doi.org/10.1016/j.yjmcc.2024.09.012. [file EMS200381-supplement-Supplementary_Material.zip › 1-s2.0-S0022282824001615-mmc3.pdf]

Ex vivo modelling of cardiac injury identifies ferroptotis-related pathways as a potential therapeutic avenue for translational medicine

Naisam Abbas (M.D., Ph.D.)[a,b,$] and Marco Bentele[a,$], Florian J. G. Waleczek[a,b], Maximilian Fuchs (M.Sc.)[b], Annette Just[a], Angelika Pfanne[a], Andreas Pich (Ph.D.)[d], Sophie Linke[a], Susanne Neumüller[a], Angelika Stucki-Koch[b], Filippo Perbellini (Ph.D.)[a], Christopher Werlein (M.D.)[e], Wilhelm Korte (M.D.)[f], Fabio Ius (M.D.)[f], Arjang Ruhparwar (M.D.)[f], Natalie Weber (M.D., Ph.D.)[a,*], Jan Fiedler (Ph.D.)[b,*], Thomas Thum (M.D., Ph.D.)[a,c,*]

[a] Institute of Molecular and Translational Therapeutic Strategies (IMTTS), Hannover Medical School, Hannover, Germany
[b] Fraunhofer Institute of Toxicology and Experimental Medicine (ITEM), Hannover, Germany
[c] Center for Translational Regenerative Medicine, Hannover Medical School, Hannover, Germany
[d] Institute of Toxicology and Core Unit Proteomics, Hannover Medical School, Hannover, Germany
[e] Institute of Pathology, Hannover Medical School, Hannover, Germany
[f] Department of Cardiothoracic, Transplantation and Vascular Surgery, Hannover Medical School, Hannover, Germany
[$] These authors contributed equally and share the first authorship
*These authors contributed equally and share the last authorship

Supplementary information

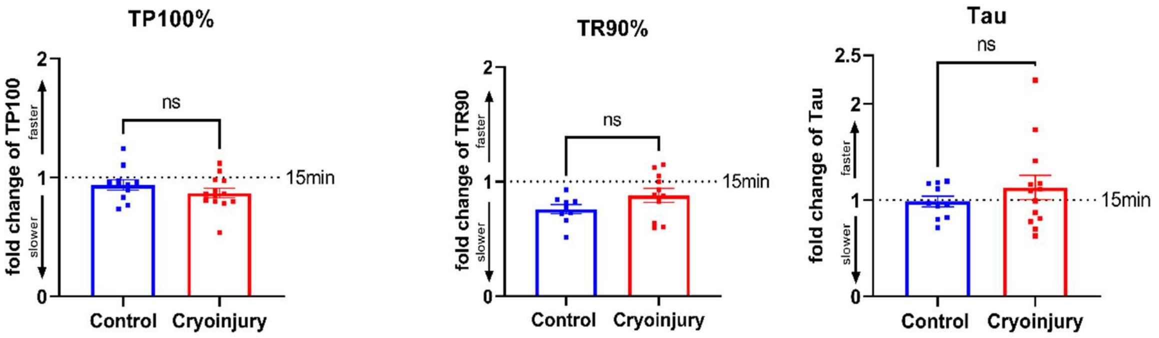

Supplementary figure 1. *Contractile parameters of rCtrl-LMS and rAHF-LMS obtained from BMCC.(*p<0.05; Student's t test; n=6). TP – time to peak; TR – relaxation time. Data are displayed as mean±SEM.*

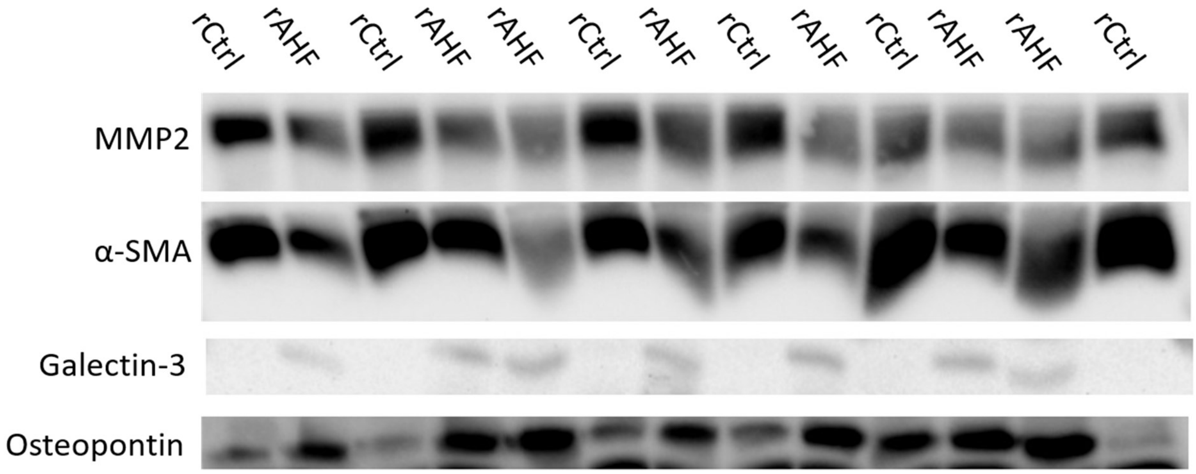

Supplementary figure 2. *Western blot analysis of secreted proteins (MMP2, α-SMA, galectin-3 and osteopontin) into the supernatant of rCtrl- and rAHF-LMS*

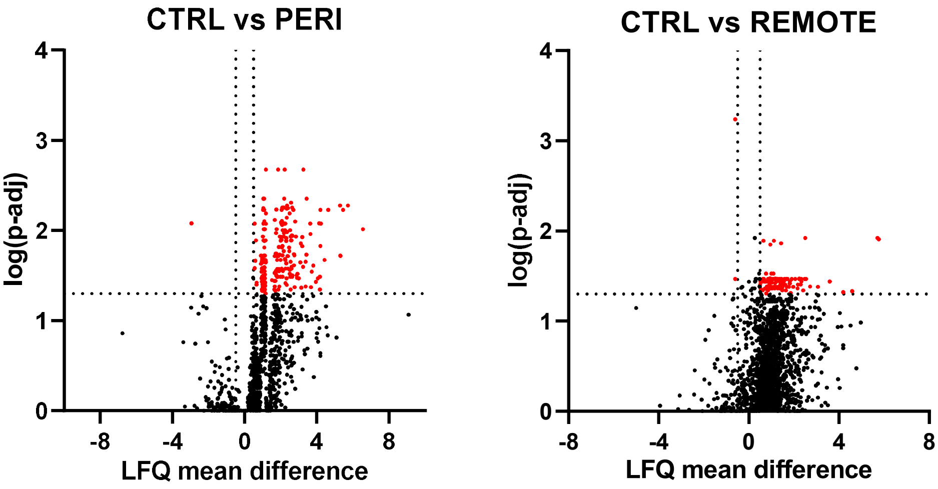

Supplementary figure 3. *Volcano plots of preoteomics dataset. Left – rCtrl vs. rAHF-Peri-injury. Right - rCtrl vs. rAHF-Remote (p-ajd<0.05; |Log_2FC|>0.7; n=3).*

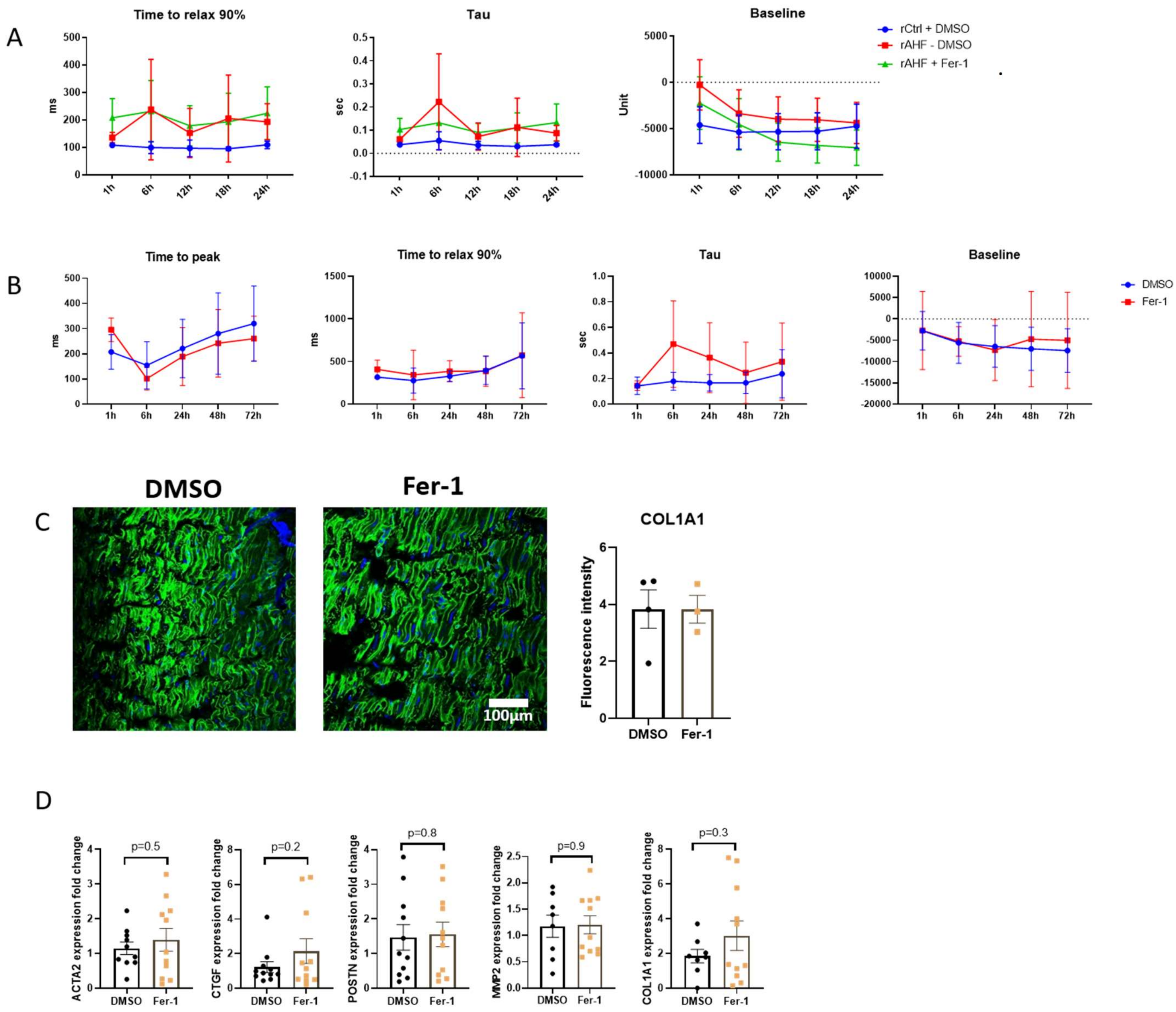

Supplementary figure 4. *(A) Time course of relaxation parameters of rat LMS (rCtrl- and rAHF-LMS) ± Fer-1 [10µM] treatment during culture in BMCC: Time to 50% relaxation, Tau, baseline (two-way ANOVA; n=4). (B) Time course of contractile parameters of human LMS (hCHF-LMS) ± Fer-1 [10µM] treatment during culture in BMCC: Time to peak, Time to 90% relaxation, Tau, baseline (two-way ANOVA; n=5-6). (C) Representative images and quantification of collagen I immunostaining in hCHFLMS ± Fer-1 [10µM] treatment (Student's t test, n=3-4). (D) RT-qPCR quantification of fibrosis-related gene expression in hCHF (Student's t test; n=11). Data are displayed as mean±SEM.*

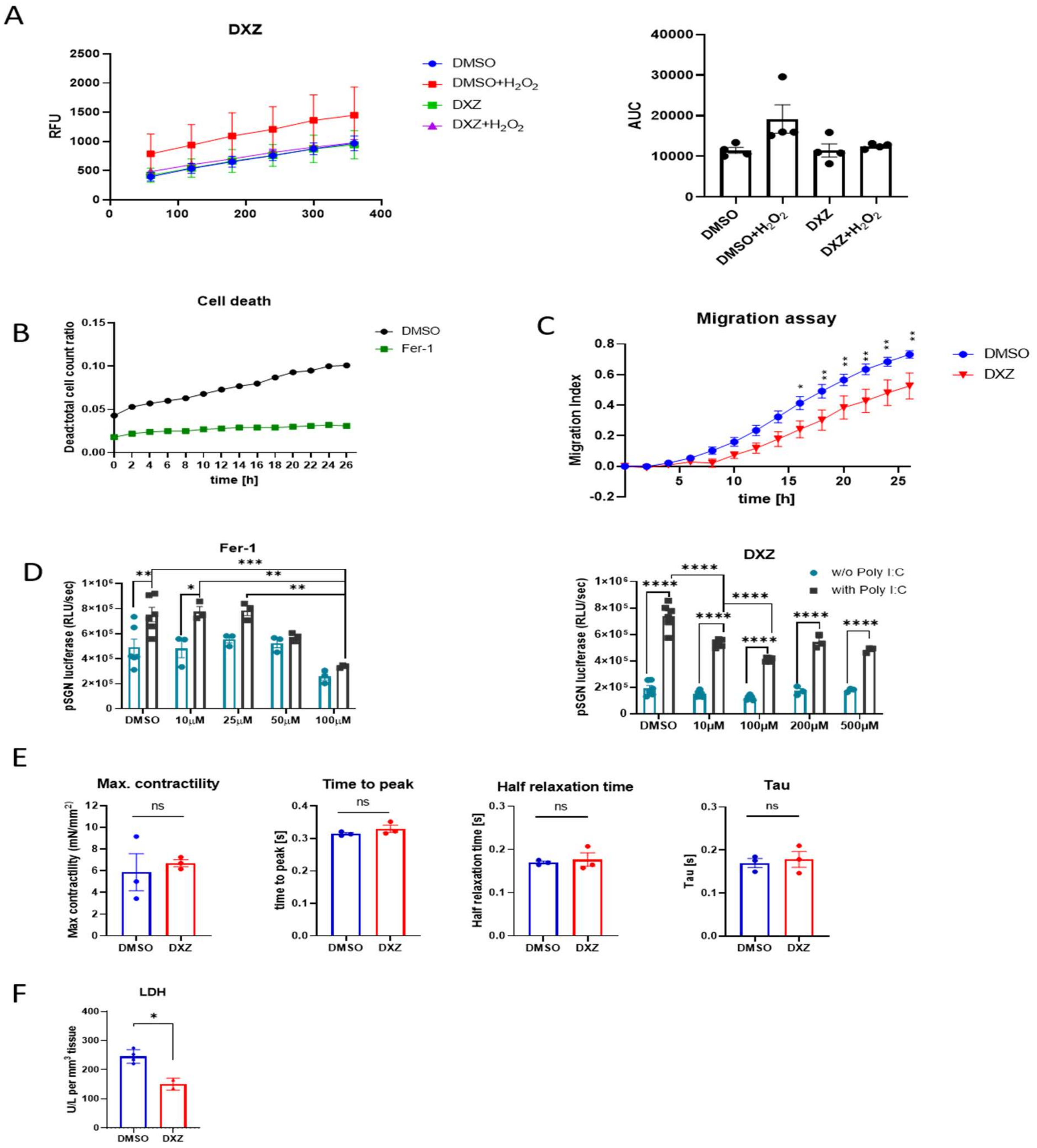

Supplementary figure 5. *(A) ROS production assay in human cardiac fibroblasts over 6h, with and without H_2O_2 stimulation. DXZ [100μMwas used to inhibit ferroptosis. Right – comparison of AUC anlysis (one-way ANOVA; n=4). (B) Cell death quantification via CellTox cytotoxicity staining over 26h. (C) Migration index over 26h – measurement were done in 2h intervals (*p<0.05, **p<0.01; two-way ANOVA; n=3). (D) NF-κB reporter assay measuring the expression of NF-κB-dependent luciferase by in HEK293 cells, with and without poly I:C stimulation. (*p<0.05, **p<0.01, ***p<0.001, ****p<0.0001; two-way ANOVA; n=3). Data are displayed as mean±SEM. (E) Force measurement of LMS in the force transducer and quantification of contractile parameters: Maximal contractility, Time peak, Half relaxation time and Tau (τ; Student's t test; n=3). Data are displayed as mean ± SEM. (F) LDH release measured from supernatants of human LMS cultured with or without DXZ). (*p<0.05, ordinary one-way ANOVA; n=3).*

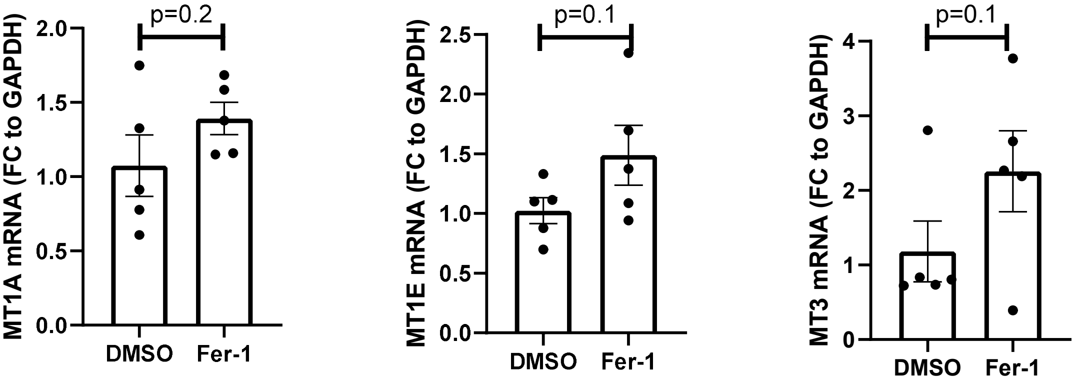

Supplementary figure 6. *RT-qPCR quantification of metallothionein gene expression in HCF cells (Student's t test; n=5). Data are displayed as mean±SEM*

Supplementary table 1*: DAPs found in proteomics analysis: rAHF-P vs. rCtrl and rAHF-R vs. rCtrl. (|log2FC|≥0.5; pajd≤0.05; n=3)*

rAHF-Peri Injury vs. rCtrl		rAHF-Remote vs. rCtrl	
Upregulated	Downregulated	Upregulated	Downregulated
RGD1565355	Cox15	Epb4.1	Commd7
Hist2h3c2;Hist1h3e	Sdhaf1	Hmox2	Cab39
Epb4.1	Ostf1	Gmfb	Smim8
Apoa1	Acaa1a;Acaa1b	Mybbp1a	LOC100912478
Ahsg	Bag5	Ctsl	Blvra
Apoh	Clic5	Parva	Smc1a
Hmox2	Mrpl39	Parl	Ehbp1l1
H3f3b;H3f3c	Cbr1	Hist2h3c2;Hist1h3e	
Hist1h4b	Plaa	Plxnb2	
Ttr	Trim28	Hnrnpa0	
F2	Khdrbs1	Rpl21	
Glg1	Clip1	Glg1	
Tmem182	Gsk3b	RGD1565355	
H2afy	Psmd5	Serhl2	
Fgg	Tollip	Nqo1	
Hpx	Smim8	Plp2	
Hist1h2bk	Blvra	Hnrnpul2	
Fn1	Ndufaf5	Prkacb	
Fgb	Bola3	Adhfe1	
M0RAV0	Cab39	Dbnl	
C3	Ehbp1l1	Gnb3	
Plp2	Smc1a	Mtnd4l	
Apoe		Bdh2	
Enpp1		Eef1e1	
Serpina1		Rhoc	
Dnajc5		Cnp	
Fga		Crip1	
Ephx1		Csrp2	
Plg		Tspan18	
Cd48		Apoa1	
Mtdh		Ddx21	
Gc		Tagln	
Tspan18		Ddx19a	
Mmgt1		Hist1h1c	
Tspan9		Ccdc127	
Parva		Gna11	

Plxnb2		Atl3	
Dhrs7b		Hist1h4b	
Gnb1		Dync1li2	
Sdcbp		Srsf1	
H2afz;H2afv		Rpl7a	
Abhd12		Arhgef1	
Icam2		Agpat3	
Trim26		Fundc1	
Tf		A0A0G2JW88	
Pcyox1l		Rer1	
Dhrs7		Cd48	
Stx7		Pcyox1l	
Rer1		Tmem261	
RT1-Bb		Icam2	
Ogn		Gnb1	
Art4		Ogn	
Mfge8		Vti1b	
Cp		Stx7	
Mtnd4l		Rcn1	
Apoa4		S100a13	
M6pr		Rcsd1	
Napepld		Cd38	
Gsn		Pfn2	
Cd38		Rnh1	
Cd34		Lyn	
Alb		M6pr	
Fbn1		Gpr107	
Chchd10		Fhl2	
Gnb3		Phospho1	
Clec10a		Gsta3	
Emcn		LOC100911683	
Cd151		Bag5	
Ppap2a		Pds5b	
Hk1		Ap2m1	
Actn2		Rpl7	
Bcl2l1;Bcl2l1-ps1		Tmx3	
S100a13		Abhd12	
Pcyox1		Rpl4	
Serpina3k		Septin-9	
Itfg3		Hist1h1e	
Fundc1		Mtdh	
Tmed7		Sco1	
Cnp		Snx12	

Lpl		Prpf19	
Cntnap3		Rpl6	
Sirpa		Numa1	
Ppap2b		Psap	
Mybbp1a		Bche	
Gpd2		Usp9x	
Enpp4		Gnaq	
Lrp1		Atp5e;LOC100361879	
Selenoi		Tmem182	
Rpl7a		Hist3h2bb	
Dbnl		Nolc1	
Tmx3		Csrp1	
Myh10		Ppp2r5c;LOC100909464	
Xpnpep2		Coq10b	
Cpne1		Fgb	
Tmed4		H2afz;H2afv	
Arhgef1		Myoz2	
Agrn		Stxbp1	
Hist1h1e		Chmp6	
Ctsl		Dhrs7	
Cd59		Cast	
Entpd2		Cdh5	
Esyt1		Hbe1	
Cd36;LOC685953		Dhrs7b	
Itga7		AI314180	
Endod1		Cd59	
Chmp6		Chpt1	
Serhl2		Cbx3	
Hist1h1c		Mxra7	
Mtfp1		Enpp1	
Lmod2		H3f3b;H3f3c	
Sptan1		Rbbp4	
Tagln		S100a4	
Ptgfrn		Denr	
Ccdc167		Dnajc5	
Cyb5a		Sqstm1	
Alpl		Flna	
Cebpzos		Mmgt1	
Gpr116		Fxyd1	
Srsf1		Dctn5	
A1m		Inpp1	
Prkacb		Myo1b	
Rps6		Kpna4	

Ca4		Abcb10	
Serpinc1		Ahsg	
Igf2r		Hpx	
Tfrc		Smc3	
Cd47		Lrp1	
Itga1		Rpl18	
Slc44a2		Ppap2a	
Aldh3a2		Thy1	
Eef1e1		Itfg3	
Rpl18		H6pd	
Nedd4l		Rpl13a	
Ccdc127		Gc	
Tmem261		Rhoa	
Nqo1		Cd36;LOC685953	
Dag1		Sirt3	
Cst3		Mfge8	
Lamp1		Sncg	
Fam210a		Psmb9	
Gpx3		Src;Fyn;Yes1;Lck;Hck	
Bcap29		Ktn1	
Pigs		Vamp3	
Cd81		Epb41l2	
Ermp1		Fam120a	
Anpep		Cox6a1	
Cr1l		Skp1	
Thy1		Gsn	
Ccdc176		Tardbp	
Eng		Tgm2	
Derl1		Gng2	
Itgav		Prkcdbp	
Entpd1		Pgm2	
Sts		Rps6	
Nt5e		Cnn3	
Surf4;Surf1		Agk	
Clec2d11		Esyt1	
Bgn		Chchd7	
Cd9		Mt-atp6	
Sypl1		Mcam	
Dpp4		Sub1	
Col6a1		Hist1h1b	
Ctsz		Rab5a	
Bche		Mtnd3	
Rpl6		Mrpl39	

Mrpl27		Smarcc2;Smarcc1	
Rtn4		Parp1	
Gpr107		Capza1	
Enpep		Ctsz	
Dcn		LOC683884	
Tspan8		Rab5b	
Atl3		Txn2	
Myh14		Hmgn2;LOC100360316	
Stom		Tpm3	
Chchd7		Syngr2	
Tmem109		Cmc2	
Ndufa11		Rrbp1	
Col6a2		Col4a2	
Des		Ctsb	
Mp68		Asph	
Tmed5		Fxn	
		Zfp692	
Pxmp2		Cox6c2	
Lamc1		Nenf	
Ddx19a		Cox5a	
Scarb2		Mpc1	
Abcb10		Sypl1	
Lman1		Mp68	
Susd2		Rps11	
Mtco3		Actb	
Lama2		Ephx1	
Rhoc		Rpl15	
Usmg5		Minos1	
Podxl		Tmed4	
Adhfe1		Hnrnpa2b1	
Fis1		Cox6b1	
Anxa3		Sirpa	
Hspg2		Vdac3	
Plgrkt		Cox6a2	
Aqp1		Add3	
Cspg4		Arf4	
Apmap		Ndufv1	
Cyb5b		Bcl2l1;Bcl2l1-ps1	
Hnrnpa0		Cmpk2	
Pecam1		Ckmt2	
Csnk2b		Ppp1r12b	
Gng2		Ndufb2	
Lama4		Ehd2	

Cox20		Cspg4	
Syngr1		Calr	
Gmfb		Actg1	
Fignl1		Ralb	
Gpc4		Septin-11	
Tmed2		Cd47	
Tmem126a		Cebpzos	
Agk		Reep5	
Synj2bp		Sdcbp	
Anxa5		Ptma	
Reep5		Clec10a	
Rcn1		Nedd4l	
Vdac3		Mtfp1	
Sco1		Sars	
Cav3		Rps26	
Pln		Cd99	
Rps11		Atp1b3	
Fbln5		Acbd3	
Myl2		Tagln2	
Itga5		Surf4;Surf1	
Lamb1		Lmf2	
Cdh13		Cox17	
Cox6c2		Rpl30	
Dctn5		Ada	
Vamp3		Fkbp1a	
Cd200		Mrpl27	
Anxa4		Myl2	
Chpt1		Ehd1	
Anxa2		Tjp1	
Selt		Ccdc176	
Minos1		Fgg	
Ndufb2		LOC100912599	
Col4a2		Gpd2	
Tmod1		Cox20	
Slc25a11		Cap1	
Bsg		Itga7	
Rtn3		Pi4ka	
Cd99		Tf	
Psmb9		Tpm4	
Anxa1		G3bp2	
Rlc-a		Lamp1	
Etfdh		Septin-8	
Pi4ka		Khdrbs1	

Fam162a		Sts	
Vti1b		Fn1	
Atp5e;LOC100361879		Cp	
Capza1		Clic1	
Capzb		LOC684828	
Bcap31		Ptrf	
Banf1		Tmpo	
Myl6		Rtn4	
Itga6		Rexo2	
Rpl21		Stx12	
Hist1h1b		Bcl2l13	
Itgb1		Hmx1	
Vdac1		Podxl	
RGD1565410;LOC100911104		LOC685596	
Phb2		Vim	
Car14		Atp5d	
Cacna2d1		Ccbl1	
Vnn1		Mtco3	
Mtnd3		Mt-Cyb	
Cox5a		Tfam	
Mtch1		Lamtor3	
Lum		Alb	
S100a10		Ndufa6	
Enpp3		Gstp1	
Rplp1		Ndufa5	
Crip1		Coq5	
Anxa7		Timm8a;Timm8a1	
Rpl10;Rpl10l		Pgrmc1	
Lnpep		Pccb	
Lamb2		Pars2	
Erlin2		Ilk	
Lmf2		Rpl10;Rpl10l	
Ghitm		Ndufv3	
Parl		Akr1c9	
Cox6a2		Ywhaq	
Mt-atp6		Aldh3a2	
Coq7		Hmg1l1;Hmgb1;Hmgb1-ps3	
Myoz2		Tmem33	
Col15a1		Taldo1	
Lamp2		Myl6	
Canx		LOC681355	
Pdcd6		Eef1a1	
Flot2		Cdnf	

Rcsd1		Strn3;Strn4	
RGD1565784		Atp5l	
Fxyd1		Sptb	
Rtn4		Bgn	
Asph		Q642A4	
Rpl30		Fahd1	
Fhl2		Lmnb1	
Ndufb7		C1qbp	
Rpl13a		Ppia	
Actn4		Ssbp1	
Gpc1		Sri	
Zfp692		Ndufa8	
Cox6a1		Esam	
Phb		Ptbp1	
Sri		Serpina1	
Sirt3		Eif3f	
Flot1		Psmb10	
Rab6a		Acaa1a;Acaa1b	
Rpl15		Pcyox1	
Mt-Cyb		Ssr4	
Csrp2		Rps16	
Cox7a2		Actr1a	
Gnaq		Slc9a3r2	
Psap		Myh10	
Bcam		Cd151	
Pgrmc1		Gimap4	
Cdh2		Tmed5	
Nrp1		Q68FZ8	
Ndufb9		Usmg5	
Tomm40		Entpd2	
Slmap		Pecam1	
Gng12		Rps2;Gm8225	
Sptbn1		Lta4h	
Tmem43		Map4	
Acta1		Tpt1	
Coa3		Actr3	
LOC684828		Fam210a	
Hnrnpul2		Rtn3	
Sqrdl		Ak2	
Mxra7		S100a10	
Atp5i		Cav1	
Cpne3		Fbln5	
LOC100911130		Clip1	

Ociad1		Derl1	
Esam		Q9R1T1	
Cav2		A0A0G2JUD4	
Flna		LOC100361144;LOC100362391	
Jagn1		Rps18	
Anxa6		Car14	
Sdhc		Cnpy2	
Atp5l		Pcca	
Tmem33		Hspg2	
Ncam1		Anxa7	
Prelp		Gbas	
Capza2		Mgll	
Mtco2		Capns1	
Dync1li2		Tspan9	
Ncstn		Fth1	
RGD1302996		Cyb5a	
Myl3		Sspn	
Coq10b		Rpl19	
LOC100361144;LOC100362391		Lmod2	
Bdh2		Fam162a	
Calr		Snx2	
LOC100363239		Rap1b	
Ndufb5		Lman2	
Rtn1		Rps24	
Ndufb10		Cat	
LOC683884		Lman1	
Apoo		Stoml2	
Lman2		Cox5b	
Ndufb11		Lactb2	
Pfn2		Hnrnpd	
Sub1		LOC100912534	
Rps26		Cyc1	
Adipoq		Ca2	
Smim12		Tln1	
Bcs1l		Rasip1	
Rps15a;Rps15al2		Ndufa9	
Atp1b3		Dpp7	
Sspn		Cbr1	
Lamtor3		Ca4	
Rap1a		Ndufb10	
Nid1		Col6a1	
Gbas		Add1	
Csnk2a1		Pnp	

Pgrmc2		Tfrc	
Rpl19		Ccdc167	
Ralb		mrpl11	
Rpl4		Ndufb7	
Napa		Rpl34;Rpl34-ps1	
Vcan		Ndufs4	
Cisd2		Got2	
Rhoa		Lamc1	
Rap1b		Timm21	
Ndufb6		Capzb	
Rab5a		Mtch1	
Asah1		Me3	
Tmem47		Rps14	
Cox4i1		Anxa11	
Csrp1		Ssb;LOC680385	
Art3		Ctsd	
Nceh1		Tmem126a	
Cisd1		Anp32b	
Apool		Nudt21	
Ndufb1		Col6a2	
Cdipt		Actn4	
S100a4		Magohb;Magoh	
Vps13a		Nfu1	
Tmem70		Anxa5	
Rps24;LOC100363469		Fga	
Ndufa8		Vdac1	
Rpl38;RGD1561636		Tmx4	
Ssr4		Cav3	
Fundc2		Idh3a	
Syngr2		Rpl27;RGD1563835	
Stt3a		Ppp3ca;Ppp3cb	
Vdac2		Sdpr	
Usp9x		Hadh	
Pon2		Psmd7	
mrpl11		LOC684270	
Ndufa4		Slc44a2	
Rps25		Elavl1	
Septin-10		Sdhaf1	
Gfer		Txn;Txn1	
Rnh1		Marcks	
Psmb10		Bphl	
Timm22		Ppif	
Ddx21		Tmed7	

Rps18		Cr1l	
Vat1		Apmap	
Anxa11		Aldh1a1	
Timm8a;Timm8a1		Tmed2	
Vdac3		Msn	
Dhodh		Nit2	
Cav1		Clic5	
Tmed10		Atp5f1	
Timm9		Vat1	
Pam16		Lamb1	
Mcam		Ddost	
Pyroxd2		Akr1a1	
Snrpd1		Ppp1ca	
Higd1a		Phb2	
LOC685596		Cd200	
Tmem256		Apoh	
Mgst3		Napa	
Ndufaf3		Sgca	
Tmem65		Dpysl2	
Ctsb		Cpne3	
Dpp7		Tpm1	
Rpl7		Sdhc	
Gypc		Anxa2	
Lyn		Uqcrh	
Atp5j2		Mtch2	
Mtco1		Gstm1	
LOC100911483;Ndufa13		Glb1	
Stoml2		Fscn1	
Hpcal1;Hpca		Smim12	
Uqcr10		Capza2	
Actg1		Trim28	
Cisd3		Ostf1	
Gna11		Anxa3	
Timm23		Prdx1	
Atp5f1		Itga1	
Cox6b1		Cfl1	
Slc25a21		Clybl	
Tmem14c		Rps12	
Gsta3		Eef1b2	
Map4		Vps13a	
Cyc1		Uqcrc1	
Mt-atp8		Actr1b	
Eif3f		Hrc	

Cpt1a		Dld	
Ptges2		Tpm1	
Mtnd4		Ppic	
Rbbp4		Emc2	
B2m		Arpc2	
Agpat3		Bsg	
LOC688684;Rpl32		Agrn	
Cap1		Lgals1	
Ndufa5		Bcap31	
Cdh5		Etfdh	
Ndufa6		Tomm40	
Timm21		Asah1	
		Hibch	
		Mdh2	
		Tomm22	
		Lamp2	
		Gk	
		Rab11a	
		Snrpd1	
		Adck3	
		Lama2	
		Plbd1	
		Arpc3	
		Ndufb9	
		Rpl11	
		Stt3a	
		Pln	
		Rap1a	
		Casq2	
		Slc25a11	
		Myh14	
		Ndufs7	
		Coq9	
		Gnai2	
		Akr1cl	
		Pcmt1	
		Itgav	
		Dag1	
		Art3	
		Gfer	
		Uqcrc2	
		Anp32a	
		Atp1b1	

		Bcap29	
		Ndufa11	
		Vdac2	
		Cycs;LOC679794	
		Sgcg	
		Cd9	
		Hsd17b10	
		Nutf2	
		Ywhae	
		Csnk2a1	
		Dysf	
		Pgd	
		Acta1	
		Glod4	
		Tspan8	

Supplementary table 2: *Supplementary table 2: Clinical information of the patients enrolled in the human heart failure LMS experiments.*

Patient	Age	Sex	Diagnosis	EF%
1	11	male	DCM	LVEDD 60
2	61	male	ICM	10
3	60	female	ICM	50
4	20	female	CHF following myocarditis	<10

DCM – dilated cardiomyopathy; ICM – ischemic cardiomyopathy, CHF – congestive heart failure

Supplementary table 3: *DEGs found in RNA seq analysis: hCHF +Fer-1 vs. hCHF DMSO (|log2FC|≥0.5; pajd≤0.05; n=4).*

hCHF: Fer-1 vs. DMSO	
Upregulated	Downregulated
MT1H	DDO
GPRC5A	LMCD1
RHCG	PDCD2L
HAS1	NPR3
HID1	DSG2
SLC3A2	AC022034.1
IL1RL1	SSX2IP
SLC7A5	VSIR
KLF4	LINC01936
BAIAP2	PLCXD3
SESN2	MTND2P28
ATF3	CAVIN4
DERL3	PKP1
DDIT3	RANBP6
MT1E	MT-ATP8
HIF1A-AS3	MT-ND2
GABRR2	MT-ND3
STX3	MT-ND1
CTH	E2F8
LSMEM1	H19
PTPRN	AC103740.1
GEM	MT-TL1
MT1F	MT-ND4L
ZNF469	RAPGEF4
ULBP1	IGF2
RND1	AL365434.1
LINC00520	MT-ATP6
CBX4	RGS5
SMG1P7	KLHL38
SOX30	MTATP6P1
CDC6	HIGD1B
UAP1L1	CA4
SCX	MT-CO1
LMNTD2-AS1	MT-CO3
SMOX	MT-ND4

LURAP1L-AS1	MT-ND5
PDIA2	CX3CL1
NR4A2	CD300LG
MAFA	GPIHBP1
ZNF425	ALDH1B1
HMOX1	FKBP5
LRP2BP	PALLD
LINC00862	SORBS1
MANF	LPCAT3
ESM1	
KCNQ1OT1	
SLC30A2	
TMEM63C	
CHAC1	
HERPUD1	
AC068580.1	
ASPRV1	
BHLHA15	
TRIM36	
LRRC15	
FAM222A	
MT1X	
ENPP1	
MMP10	
FOSB	
C2CD4A	
AC144831.1	
VASN	
RETREG1	
NR1D2	
FAM107B	
ZSCAN5A	
MXD1	
BEST1	
SRPK3	
HSPA1A	
PMEPA1	
CEBPG	
MTHFD2	
ANKRD37	
SLC7A11	
KRT8P12	
TP53INP2	

ZBTB21	
NFKBIZ	
FAM117A	
MAFB	
EIF2AK3	
GCLM	
SHMT2	
